# Long-term mental health of war-refugees: a systematic literature review

**DOI:** 10.1186/s12914-015-0064-9

**Published:** 2015-10-28

**Authors:** Marija Bogic, Anthony Njoku, Stefan Priebe

**Affiliations:** Unit for Social and Community Psychiatry, WHO Collaborating Centre for Mental Health Services Development, Queen Mary University of London, E13 8SP London, UK; Fredericton OSI Clinic, NB, Canada

**Keywords:** Refugees, War trauma, PTSD, Depression, Anxiety, Risk factors

## Abstract

**Background:**

There are several million war-refugees worldwide, majority of whom stay in the recipient countries for years. However, little is known about their long-term mental health. This review aimed to assess prevalence of mental disorders and to identify their correlates among long-settled war-refugees.

**Methods:**

We conducted a systematic review of studies that assessed current prevalence and/or factors associated with depression and anxiety disorders in adult war-refugees 5 years or longer after displacement. We searched Medline, Embase, CINAHL, PsycINFO, and PILOTS from their inception to October 2014, searched reference lists, and contacted experts. Because of a high heterogeneity between studies, overall estimates of mental disorders were not discussed. Instead, prevalence rates were reviewed narratively and possible sources of heterogeneity between studies were investigated both by subgroup analysis and narratively. A descriptive analysis examined pre-migration and post-migration factors associated with mental disorders in this population.

**Results:**

The review identified 29 studies on long-term mental health with a total of 16,010 war-affected refugees. There was significant between-study heterogeneity in prevalence rates of depression (range 2.3–80 %), PTSD (4.4–86 %), and unspecified anxiety disorder (20.3–88 %), although prevalence estimates were typically in the range of 20 % and above. Both clinical and methodological factors contributed substantially to the observed heterogeneity. Studies of higher methodological quality generally reported lower prevalence rates. Prevalence rates were also related to both which country the refugees came from and in which country they resettled. Refugees from former Yugoslavia and Cambodia tended to report the highest rates of mental disorders, as well as refugees residing in the USA. Descriptive synthesis suggested that greater exposure to pre-migration traumatic experiences and post-migration stress were the most consistent factors associated with all three disorders, whilst a poor post-migration socio-economic status was particularly associated with depression.

**Conclusions:**

There is a need for more methodologically consistent and rigorous research on the mental health of long-settled war refugees. Existing evidence suggests that mental disorders tend to be highly prevalent in war refugees many years after resettlement. This increased risk may not only be a consequence of exposure to wartime trauma but may also be influenced by post-migration socio-economic factors.

## Background

Worldwide there are over 19 million refugees, most of whom were displaced because of war and other organized violence [1]. The majority of refugees stay in the recipient countries for years or even decades. It is estimated that by the end of 2014 nearly a half of the world’s refugees will have lived in protracted refugee situations, which means that they have been in exile for 5 years or longer without immediate prospects for durable solutions. On average, however, a refugee spends more than 20 years in exile before he or she can go back home or find another solution [1]. Refugees’ mental health often presents a challenge to clinicians and policy makers of the recipient countries.

Evidence from community studies amongst recently resettled refugees suggests that refugees have higher rates of mental disorders, in particular depression, PTSD and other anxiety disorders, than those usually found in the non-war affected general population [2, 3]. Several longitudinal studies amongst recently resettled refugees have indicated that post-traumatic stress reactions may persist and even increase over time [4–6], at least during the immediate period after war trauma and resettlement. This increased vulnerability has been linked to both pre-migration experiences, in particular exposure to war trauma [7, 8], and post-migration conditions and stressors refugees often face in a new country, including separation from family, difficulties with asylum procedure or even detention, unemployment, inadequate housing, and issues related to acculturation [9, 10].

Whether the refugees’ increased risk of adverse mental health persists after the initial period of resettlement is unclear since there is a paucity of comparable data for long-settled refugees and the few studies that have been undertaken present an inconsistent picture. Whilst some studies reported a gradual improvement in symptoms over a period of a decade, to the point where prevalence rates of mental disorders were lower than in the general population of the host country [11, 12], other studies found prevalence rates substantially higher than those in the general population [13–15]. Previous systematic reviews and meta-analyses evaluating mental health of refugees (including those recently resettled) have all indicated a reduction in risk for mental disorders as the length of time since displacement increases [2, 8, 9]. However, these reviews did not specifically report findings for refugees with a longer duration of displacement [8, 9, 16, 17], mostly assessed studies of recently resettled refugees [9, 17] where rates would be expected to be higher, focused only on refugees in Western countries [2], and confined their findings to PTSD, depression or a generic effect size index of psychological distress derived from heterogeneous outcome measures across studies [8, 9, 17]. Thus, a systematic review focusing specifically on long-term mental health outcomes of war refugees worldwide is warranted. Understanding the long-term mental health of refugees is essential for guiding the health policies of recipient countries aimed at promoting long-term mental health of refugees [15, 18, 19].

The current review investigated whether mental disorders in war refugees persist beyond the immediate period after war trauma and resettlement by focussing on studies assessing mental disorders and factors associated with these disorders among long-settled war refugees, including those residing outside Western countries. The specific aims of the present review were to:

i. assess the prevalence of depression and anxiety disorders among community samples of adult war refugees 5 years or longer after displacement;
ii. assess the influence of methodological and contextual factors on the prevalence rates found in the studies; and
iii. identify pre-migration and post-migration factors associated with the prevalence of these disorders.

## Methods

### Search strategy

Five electronic databases (CINAHL, PsychINFO, EMBASE, Medline and PILOTS) were searched from their inception to October 2014, using a combination of keywords “refugee”, “mental health”, “depression”, “PTSD”, “anxiety”, “long-term” and various synonyms as search terms. Key search terms are presented in Table 1.

**Table 1:** Key search terms and combination operators

Key search terms
Refugee: refugee, displaced persons, asylum seeker, stateless person, war survivor, conflict survivor, war victim, exile, OR uprooted person
Mental health: mental health, mental disorders, mental illness, psychiatric symptom, well-being, psychopathology, clinical or psychiatric or psychological outcome, psychiatric morbidity or disability, psychological morbidity or disability, adjustment problem, distress, psychological stress, posttraumatic stress, stress, PTSD, anxiety, depression, depressive disorder, traumatic reaction, psychological reactions OR psychiatric reactions
Long-term: chronic, enduring, prolonged, persistent, ongoing, continuing, longitudinal OR durable
Combination operators
Long-term refugee mental health: Refugee (all terms) AND mental health (all terms) AND long-term (all terms)

Articles with refugees were identified using MeSH and text keywords for “refugees”, whilst “mental health” content in articles was identified using MeSH and text keywords for “mental health” and specifically for “depression”, “anxiety”, and “PTSD”. In order to focus the search terms on the studies with long-settled refugees, the search strategy also included the term “long-term”. Text keywords were used to identify articles indexed by “long-term”. These terms were adapted for each database. For example, terms for Medline were: (stateless person; asylum seeker; conflict survivor; displaced person; refugee*; war victim; exile; uprooted; war survivor) AND (anxiety; anxiety disorder*; ptsd; posttraumatic stress disorder; posttraumatic stress; distress; psychological stress; adjustment problem; psychological adaptation; adjustment disorder*; quality of life; well being; psychiatric outcome; psychological outcome; clinical outcome; psychopathology; depression; major depressive disorder; mental illness; mental health; mental disorder*; mental health; psychiatric symptom; psychiatric reactions; psychological reactions; psychiatric morbidity; psychiatric disability; psychological disability; traumatic reaction) AND (ongoing; prolonged; persist*; enduring; chronic; long term; longitudinal; durable).

The references of included articles were then screened to identify any further relevant papers, as were the contents of systematic reviews identified through searches [2, 3, 7–10, 16, 17, 19]. In addition, data from unpublished studies or articles in press were sought by informally contacting experts in the field.

### Inclusion and exclusion criteria

Studies were included in the review if they: i) investigated a community adult sample of war refugees 5 years or longer after displacement; ii) had a sample larger than 30 participants; and iii) reported quantitative estimates of depression, PTSD, and/or anxiety or reported on their associative factors. There were no restrictions regarding the language. Populations were identified as being war refugees if they migrated from a country subjected to armed conflict. A minimum sample size of 30 was chosen to achieve a representative distribution in line with the central limit theorem [20]. To exclude the effects of age-differential vulnerabilities for trauma-related disorders, studies were excluded if the majority of the sample were younger than 12 years at the time of the last war-related traumatic event [21–23]. Case reports, qualitative studies or studies assessing clinical samples were excluded. Qualitative and quantitative research is grounded in different methodological paradigms and reviewing qualitative studies would have required a different methodology. Therefore, the inclusion of qualitative studies was beyond the scope of this review. Furthermore, these studies were excluded because the intention of the review was to perform a quantitative meta-analysis. Inclusion of clinical samples – that is inpatient, outpatient or help seeking groups - could potentially bias results and lead to an overestimation of mental health problems in refugees. When different population groups than those of interest (e.g. immigrants) were included in a study, studies were included only if refugee data were reported separately. Where multiple publications presented identical data from the same study, the most informative publication of the study was included and the other related articles were consulted for additional information.

### Data extraction

Following a fixed protocol two reviewers (M.B. and A.N.) independently assessed all citations for possible inclusion in the review. For every eligible study, information was extracted on study characteristics (e.g. methods, publication year), participant characteristics (e.g. socio-demographic data, trauma history), and statistical outcome information (prevalence rates and associated factors). Any discrepancies between researchers were resolved by discussion. The reviewers contacted 11 study authors to obtain additional data, of which ten responded and five provided the information.

### Assessment of methodological quality

Currently there is no consensus on how to assess either the quality of observational studies, in particular cross-sectional studies, or the impact of the study quality on the meta-analysis [24, 25]. This is even more the case for cross-sectional studies in refugee research, which is characterized by sampling and assessment challenges [2]. For example, language used to conduct assessment has been identified as an important criterion in determination of prevalence rates [2]. A search of literature by the current authors did not identify a validated quality assessment tool appropriate for the purposes of this study or pre-existing quality assessment tool that had been applied in previous systematic reviews of refugees’ mental health. Therefore, a five-point-quality-appraisal tool was devised specifically for this study. The assessment of the methodological quality of individual studies included in the review was guided by general guidelines for assessing prevalence studies [26] and key quality criteria identified in previous reviews of refugees’ mental health [2, 8, 9]. The criteria developed were as follows:

1. The sampling
  - The use of random or inclusive sampling (non-random = 0, random or inclusive = 1)
  - The sample size if non-random sampling (<200 = 0, ≥200 = 1);
2. The sample representativeness i.e. was the sample frame a true or close representation of the target population (not representative = 0, representative = 1);
3. The response rate (<60 % = 0, ≥60 % = 1);
4. The use of validated and reliable measurements (valid and reliable measure not used = 0, valid and reliable measure used = 1);
5. The interview was conducted in the interviewees’ native language, as opposed to through an interpreter (through interpreter = 0, native language = 1).

The first three of these criteria relate to the minimisation of sample selection bias, whilst the remaining two criteria relate to assessment validity of the studies. A cumulative score was calculated for each study. The resulting quality scores ranged from 0 to 5, with lower quality studies receiving a score of 0–3 and high quality studies receiving a score of 4–5.

### Data synthesis and analysis

The prevalence estimates of mental disorders were calculated with 95 % confidence intervals (CIs) in each study and in the pooled data. Prevalence rates were for current diagnoses with the exception of studies reporting 1-year prevalence as assessed by the Composite International Diagnostic Interview (CIDI; [27]). For longitudinal studies [11, 28], only the last assessment point prevalence was extracted for inclusion in the analyses to insure independence between studies. Of note, the earlier assessment points of the included longitudinal studies were outside the required inclusion criteria of 5 years or longer after the displacement. One study [29] followed up a small subsample (39 %) of the original study sample [30] three months to three-and-a- half years later. However, this study did not report on prevalence rates but only on predictors of PTSD symptoms. These findings were reported in the narrative review.

For anxiety disorders, the findings were reported separately for each disorder i.e. PTSD, Generalized Anxiety Disorder (GAD), Social Phobia, Panic Disorder, Obsessive Compulsive Disorder (OCD) and Agoraphobia. Additionally, findings were reported also separately for studies that used self-reported screening assessment tools, mostly the HSCL-25 anxiety subscale, to assess the presence of anxiety symptoms. While these tools do not provide a diagnosis, they are used to identify possible clinical cases of unspecified anxiety disorder based on the established cut-off score. The symptoms assessed with this scale most closely refer to GAD (e.g., [31, 32]), but are also highly concordant with other anxiety disorders (e.g., [33, 34]) i.e. it is a nonspecifc measure that overlaps with many anxiety disorders. Therefore, those identified as potential clinical cases may include individuals suffering from one or more different types of anxiety disorders. Because of the large overlap with different anxiety disorders, it was decided to assess unspecified anxiety disorder as a separate mental health outcome representing those potentially symptomatic of any anxiety disorder.

Estimates of mental disorders reported in the included studies were first described narratively. Heterogeneity is common in meta-analysis of epidemiologic data and probably should be viewed as the expectation rather than the exception [35, 36]. Statistical heterogeneity is a consequence of clinical or methodological heterogeneity, or both, among the studies (e.g., [36]). In the presence of high statistical heterogeneity, simply combining the results into one overall estimate may be misleading and, instead, the main focus of the meta-analysis should be on trying to understand clinical and methodological sources of heterogeneity [37–40].

The prevalence of depression and anxiety disorders was assessed across studies and the overall between-studies heterogeneity was assessed to determine if a meta-analysis providing a single summary prevalence estimate was appropriate. Due to inherent clinical and methodological differences between the studies assessed, a random effects model was planned as this assumes that the study and participant characteristics are not identical across studies, and that prevalence rates may vary accordingly. The model assumes therefore that there is a distribution of “true” effect sizes rather than a single true effect, and aims to estimate the mean of this distribution of true effect sizes [41]. The degree of statistical heterogeneity between studies was explored using the *Q* and *I*^*2*^ statistics. The *Q* statistic tests the null hypothesis that all studies share a common effect size with a minimal dispersion of the effect sizes across studies. The *I*^*2*^ statistic quantifies the amount of dispersion across the effect sizes and displays the percentage of observed variance between studies that is due to real differences in effect sizes. Unlike the *Q* statistic, it is not sensitive to the number of studies considered [41, 42]. *I*^*2*^ values range from 0 to 100 %, with values of 25, 50, and 75 % tentatively suggested as indicating low, medium, and high heterogeneity, respectively [43].

Possible sources of heterogeneity between studies were investigated by subgroup analysis [42–44]. When five or fewer primary studies were available, an overall statistical heterogeneity index was reported, while no further analyses were conducted. Where studies numbered six or greater, limited subgroup analyses were conducted. Several potential sources of heterogeneity were pre-specified in the protocol, and these included: region of origin, host country region, gender, displacement duration, time since resettlement, sample size, sampling method, diagnostic method, interviewer language, study quality and publication date. The degree of heterogeneity between the subgroups was assessed using the *Q*_*between*_ statistic within a random effects model. Forest plots were used to visualize the extent of heterogeneity among studies.

The possibility of publication bias was assessed using the Begg-Mazumdar adjusted rank correlation test and Egger’s linear regression method. All subgroup analyses were performed using Comprehensive Meta Analysis Version 2.0 (Biostat; Englewood, New Jersey, USA).

### Narrative synthesis of factors associated with mental disorders

Two reviewers (M.B. and A.N.) independently extracted statistical data on all risk factors considered for univariate or multivariate analyses in each study. Owing to a limited number of studies reporting on comparable sets of risk factors (i.e. many risk factors were examined only by a single study) and the heterogeneity in reported effects, a formal meta-regression analysis was deemed inappropriate [41]. Instead, a method of vote-counting was used to determine the number of statistically significant (positive/negative) and non-significant associations for each factor across studies [45, 46]. An association between a factor and a mental disorder was considered as significant if the statistical significance level was *p* < 0.05. Based on the percent of findings supporting the association (i.e. number of studies supporting the expected association divided by the total number of associations for the given factor), the factor was classified as: no association (0–33 %); indeterminate/inconsistent (34–59 %); and positive association or negative association (60–100 %) [45, 46]. In order to exclude incidental findings from single studies, for each of the outcomes (depression, PTSD, and unspecified anxiety) a risk factor was included in the narrative synthesis only if it had been studied in at least three studies for that disorder. The findings were described narratively.

## Results

**Fig. 1:**
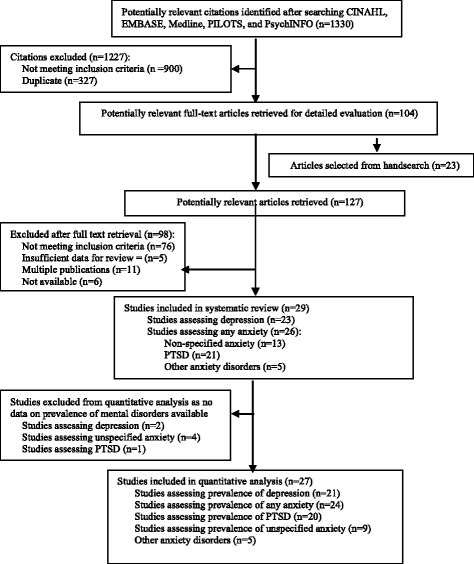
Flowchart of study selection

The systematic review process is shown in Fig. 1. Twenty-nine studies [11, 13–15, 28, 30, 47–69] met the inclusion criteria, including 23 studies assessing depression and 26 assessing anxiety. Only one eligible study published in a language other than English was identified, but another publication of the same study in the English language was already included in the review [47]. Thus, all articles included in the review were published in English.

### Study and baseline characteristics

Study characteristics are shown in Table 2. In total, the 29 studies assessed 16,010 adult war refugees (depression *n* = 12162, anxiety *n* = 11742). One study reported on unspecified anxiety and depression as a single factor since the two were highly correlated (i.e. no person had anxiety or depressive disorder without having the other) [48]. The findings from this study are reported for both depression and unspecified anxiety disorders. Four studies assessed single gender refugee samples [14, 49–51]. Across the remaining 25 studies there were slightly higher numbers of women (6044) than men (5702) in the sample. Studies were predominantly published in 2000s (*N* = 22). Refugee samples were from five main regions: Southeast Asia (65 % of refugees), Sub-Saharan Africa (21 %), Europe (11 %), Middle East (<2 %) and Central America (<2 %). Fifteen studies were undertaken in the United States, seven in Europe, four in other Western countries and four in refugee camps in non-Western countries. Studies varied in the time between leaving home and assessment, with the assessment being completed between five and 22 years after displacement (median = 9 years). Most studies were conducted within the first 10 years of the resettlement in the study country (*N* = 22; median = 8.8 years).

**Table 2:** Characteristics of studies assessing long-term mental health of war refugees

Author(s)	Refugee population (host)	Displacement duration, yrs.	Sample selection (response rate %)	Measures	Measures reliability and validity	Prevalence	Statistically significant risk factors
Beiser & Hou (2001)	608 Southeast Asian (Vietnamese Chinese, Vietnamese and Laotians) (Canada)	>10 of residence	random and non-random (95 %; follow-up = 45 %)	Self-report developed for the study	Developed for the study. No data reported.	Depression = 2.3 %	DEPRESSION
							Univariate: depressive symptoms experienced early in the process of resettlement
						(decreased over 10-year period)	
				-Depression			Multivariate: depressive levels experienced early in the process of resettlement; unemployment or unstable employment (for men); poor English language proficiency (for women and those who did not become engaged in the labour market during the earliest years of resettlement)
Bhui et al. (2003)	180 Somali (UK)	8 of residence	random (49 %)	SCQ and HSCL	Reported as previously validated in other refugee groups (data not reported).	Anxiety and Depression = 23 %	ANXIETY AND DEPRESSION
							Univariate: separated or widowed, being retired or unemployed, taking medication, not having declared asylum on entry, not having a conflict with immigration on arrival, less time in the UK (<7 yrs), shortage of food, without shelter, being lost, kidnapped, brainwashed
							Multivariate (controlling for age, gender, conflict with immigration, detention on entry, declaration of seeking asylum on entry, current asylum status, income, current employment status, employment status when in Somalia, accommodation type, cigarette smoking, alcohol use, drug use and number of residence years): number of traumatic events, shortage of food and being lost, having no combat experience
Birman & Tran (2008)	212 Vietnamese (USA)	11.5 of residence	Non-random (−)	HSCL–25	Reported as previously validated in this refugee group (data not reported).	Depression = 20.8 %	DEPRESSION
				-Depression		Anxiety = 20.3 %	Univariate: lower satisfaction with social support (by like-ethnic friends and one’s spouse), alienation, lower life satisfaction
				-Anxiety			
					In this sample, Cronbach’s alpha: Anxiety = 0.93		
							Multivariate: lower satisfaction with social support (by like-ethnic friends and one’s spouse)
					Depression = 0.94.		
							ANXIETY
							Univariate: females, poor English language, exhibiting more Vietnamese behaviour
							Multivariate: females, pre-migration trauma, exhibiting more Vietnamese behaviour
Blair (2000)	124 Cambodian (USA)	8.1 of residence	random (83 %)	NIMH DIS	NIMH DIS: Reported as previously validated in other non-refugee cultures (data not reported).	Depression = 51 %	DEPRESSION
				-Depression		GAD =14 %	Univariate: Pre-migration – higher number of war traumas, loss of immediate family member (a parent, a sibling or a child), higher number of problems while trying to escape, being separated from family while in refugee camp; resettlement – higher number of resettlement stressors, financial stress (lower rates of working outside the home, lower income, receiving welfare)
				-Anxiety		Social phobia = 27 %	
				-Social phobia		Panic disorder = 7 %	
				-Panic dis.		PTSD = 45 %	
						Comorbidity: PTSD and Depression = 71 %	
				DICA-R			
				-PTSD	DICA-R: Reported as previously validated in other non-refugee cultures (data not reported).	PTSD and Social Phobia = 32 %	
							PTSD
							Univariate: Pre-migration – higher number of war traumas, loss of immediate family member (a sibling), being separated from family while in a refugee camp, higher number of problems while trying to escape, higher number of incidents of abuse; resettlement – higher number of resettlement stressors
Bogic et al. (2012)	854 refugees from former Yugoslavia (Germany, Italy, and the UK)	9.3 of residence	random and non–random	MINI	Reported as previously validated in other non-refugee cultures (data not reported).	Depression = 34.3 %	DEPRESSION
				-Depression		PTSD = 33.1 %	Univariate: female, older age, lower education, higher number of war and post-war traumatic events, shorter time since war trauma, more migration stressors, unemployment, not feeling accepted by host country, poor host language fluency, temporary residence status, residing in Germany or the UK compared to Italy
				-PTSD		GAD = 8.7 %	
				-GAD			
				-Social phobia		Social phobia = 6.4 %	
				-Panic disorder		Panic disorder = 10.0 %	
				-Agoraphobia			
						Agoraphobia = 18.6 %	
				-OCD			
						OCD = 4.8 %	
						Comorbidity:	
						PTSD and Depression =65.1 %	
						GAD and PTSD = 43.2 %	
						Social Phobia and PTSD = 15.9 %	
						GAD and depression = 50.7 %	Multivariate: older age, lower education, higher number of war-related traumatic events, shorter time since war trauma, more migration stressors, unemployment, not feeling accepted by host country, temporary residence status, residing in Germany or the UK compared to Italy
							PTSD
							Univariate: older age, lower education, higher number of war and post-war traumatic events, longer time since war trauma, more migration stressors, unemployment, not feeling accepted by host country, poor host language fluency, temporary residence status, residing in Germany or the UK compared to Italy
							Multivariate: older age, lower education, higher number of war and post-war traumatic events, not having a combat experience, more migration stressors, temporary residence status, residing in Germany vs. Italy or the UK
							ANXIETY (any anxiety disorder)
							Univariate: female, older age, lower education, higher number of war and post-war traumatic events, not having a combat experience, more migration stressors, unemployment, not feeling accepted by host country, poor host language fluency, temporary residence status, residing in Germany or the UK compared to Italy
							Multivariate: lower education, higher number of war and post-war traumatic events, not having a combat experience, more migration stressors, not feeling accepted by host country, temporary residence status
Buseh et al. (2000)	50 Liberian males (USA)	8.6 of residence	non–random (−)	CES–D	Reported as previously validated in other non-refugee cultures (Cronbach’s alpha =0.85).	Depressive Mood =60 %	DEPRESSION
				-Depression			Univariate: more acculturative stress; CES–D subscales depressed affect, somatic/retarded activity, and interpersonal affect were positively correlated with all items on acculturative stress; CES–D subscale positive affect less perceived hate, less perceived cultural shock and lower overall acculturative stress
					In this study, Cronbach’s alpha = 0.91.		
Carlson & Rosser–Hogan (1994)	50 Cambodian (USA)	9.9 since leaving home country (5.4 of residence)	random (100 %)	HSCL–25	HSCL-25: Reported as previously validated in this refugee culture (test-retest reliability = 0.89; specificity = 0.73 and sensitivity = 0.88).	Depression = 80 %	DEPRESSION, ANXIETY, PTSD, Univariate: higher number of traumatic events
				-Depression		PTSD = 86 %	
				-Anxiety		Anxiety = 88 %	
				PCL–C –amended			
				-PTSD			
					PCL-C: Validated in other cultures (not reported in the study).		
					In this study, test-retest reliability = 0.85.		
Caspi et al. (1998)	161 Cambodian (USA)	7 of residence	random (78 %)	HSCL–25	HSCL-25 and HTQ validated in this culture (not reported in the study).	Not reported	DEPRESSION, ANXIETY AND PTSD
				-Depression			
							Multivariate: no relationship observed with child loss
				-Anxiety			
				HTQ			
				-PTSD			
Chung & Kagawa–Singer (1993)	2180 Southeast Asian (Cambodian, Laotian, and Vietnamese) (USA)	5.9 of residence	random (−)	HOS	Reported as previously validated in other non-refugee cultures (data not reported). In this study, Cronbach’s alpha for depression = 0.85 and for anxiety = 0.89.	Not reported	DEPRESSION
				-Depression			Univariate: Cambodians (followed by Lao and Vietnamese)
				-Anxiety			
							Multivariate (controlling for ethnicity and number of years in the USA): higher number of pre-migration traumatic events and female gender; for the >5 yrs in the USA: female gender, higher number of traumatic events, longer time in refugee camp, longer time in the USA, unemployment, low family income, poor English, being Vietnamese (compared to Cambodians)
							ANXIETY
							Univariate: Cambodians (followed by Lao and Vietnamese)
							Multivariate: Regardless of ethnicity and years in the USA – higher number of traumatic events, female gender, older age, receipt of public assistance, lower family income, poor English, being Lao; for refugees >5 yrs in the USA: female gender, older age, longer in the USA, higher number of traumatic events, longer time in refugee camp, unemployment, receiving public assistance, low family income, low English proficiency, Lao, Cambodian
Craig et al. (2008)	126 Bosnian (USA)	9 of residence	random (25.2 %)	MHI	MHI and PSDS reported as previously validated in other non-refugee cultures (MHI: Cronbach’s Alpha >0.80, test-retest reliability > 0.58;	Depression = 31.7 %	DEPRESSION
				-Depression		PTSD = 66.6 %	Univariate: females, older age, lower education
				-Anxiety		Anxiety = 40.5 %	
				PSDS			
				-PTSD			PTSD
							Univariate: older age, lower education
							ANXIETY
							Univariate: older age, lower education
					PSDS Cronbach’s alpha = 0.94–0.98, test-retest reliability 0.69–0.72).		
					In this study, MHI Cronbach’s alpha for anxiety = 0.95 and for depression = 0.96;		
					PSDS Cronbach’s alpha =0.97.		
D'Avanzo & Barab (1998)	175 Cambodian females (USA and France)	≥5 of residence	non–random (−)	HSCL–25	Reported as previously validated in other non-refugee cultures (test-retest reliability for depression = 0.82, anxiety = 0.84, sensitivity = 0.93 and specificity = 0.76 for either).	Depression = 73.7 % (France = 85.3 %; USA = 65 %)	DEPRESSION
				-Depression			Univariate: refugee women in France were more likely to be symptomatic of depression
				-Anxiety			
						Anxiety = 81.3 % (France = 85 %; USA = 79 %)	
					In this study, Cronbach’s alpha for depression = 0.86 and anxiety = 0.86.		
Delic-Ovcina (2010)	637 Bosnian males (USA)	6–16 (97.7 % ≥ 8 years) of residence	non–random (−)	IES	Reported as previously validated in this refugee culture (data not reported).	PTSD = 76.5 %	PTSD
				-PTSD			Univariate: older age, married, lower education, perceived poor general health, recentness of dental visit, higher frequency of smoking, lack of physical activity, no health care coverage and insufficient funds for health care services
					In this study, Cronbach’s alpha = 0.96.		
Gerritsen et al. (2006)	178 Afghan, Iranian and Somali (Netherlands)	8.8 of residence	random (59 %)	HSCL–25	Reported as previously validated in other refugee cultures (data not reported).	Depression = 29.3 %	(sample includes 232 recently arrived refugees (M = 5.6 yrs.)
				-Depression			
				-Anxiety			
				HTQ		PTSD = 10.6 %	PTSD AND DEPRESSION/ANXIETY
				-PTSD			
						Anxiety = 27.7 %	Multivariate: asylum seeker (but not for PTSD) being from Iran or Afghanistan; female, higher number of traumatic events, higher post-migration stress, less social support
Hinton et al. (1998)	3401 Vietnamese males (USA)	8–11 of residence	random (85–96 %)	HSCL–D	Reported as previously validated in this refugee culture (sensitivity = 0.86 and specificity = 0.96).	Depression = 8.8 %	DEPRESSION
				-Depression		(San Francisco = 9.8 %	Univariate: older age and veteran (in particular for those at the San Francisco and Santa Clara sites), less educated, poorer English proficiency, more recently arrived, poorer, unemployed or disabled, re-education camp survivors
						Santa Clara = 8.2 %	
						Houston = 8.6 %)	
							Multivariate: unemployed or disabled, veterans, poorer English proficiency, income below poverty line, living in Houston
Hollifield et al. (2006)	252 Kurdish and Vietnamese (USA)	Kurds = 7.0 and Vietnamese 7.8 of residence	non–random (−)	HSCL–25	HSCL-25: Reported as previously validated in other refugee cultures (data not reported).PSS-SR: Reported as previously validated in other non-refugee cultures (Cronbach’s alpha = 0.91, test-retest reliability = 0.74). In this study, Cronbach’s alpha =0.95.	Depression = 38.9 %	DEPRESSION, PTSD, ANXIETY
				-Depression		PTSD = 31.3 %	
				-Anxiety		Anxiety = 25.0 %	Univariate: higher number of war-related traumatic events
				PSS-SR			
				-PTSD			
							Multivariate: higher number of war-related traumatic events
Hunt & Gakenyi (2005)	69 Bosnian (UK)	5–8 since war trauma	non–random (69 %)	IES–R	Validated in this culture (not reported in this study). In this study, Cronbach’s alpha =0.83	PTSD = 77 %	Not reported
				-PTSD		(IES–R > 45)	
Jaranson et al. (2004)	1134 Ethiopian (Oromo and Somali) (USA)	7.5 since leaving home country (3.4 of residence)	non–random (97 %)	PCL–C	Reported as previously validated in other non-refugee cultures (data not reported).In this study, Cronbach’s alpha =0.93.	PTSD = 13 %	PTSD
				-PTSD			Univariate: being exposed to torture
							Multivariate: male, being Oromo, change in religious practices since migration, higher number of traumatic events, exposure to torture
Kolassa et al. (2010)	444 Rwandans (refugee camp in Uganda)	~13 since war trauma	random (−)	PDS	Reported as previously validated in this refugee culture (test-retest reliability = 0.93). In this study, test-retest reliability = 0.87,	PTSD = 49.5 %	PTSD
				-PTSD			Multivariate: higher number of traumatic events
					sensitivity = 0.87 and specificity = 0.86.		
Marshall et al. (2005)	490 Cambodian (USA)	20–22 of residence	random (87 %)	CIDI	Reported as previously validated in other non-refugee cultures (data not reported).	Depression = 51 %	DEPRESSION
				-Depression		PTSD = 62 %	Univariate: poor English language, retired or disabled, unemployed, below federal poverty level, older age, higher number of pre–and post-migration traumatic events
				-PTSD		Comorbidity of PTSD and depression = 71 %	
						Comorbidity of depression and PTSD = 86 %	
							Multivariate: (adjusted for age, gender, year of immigration, and pre- and post-migration trauma exposure): older age, higher number of pre- and post–migration traumatic events
							PTSD
							Univariate: older age, males, poor English language, retired or disabled, unemployed, below federal poverty level, higher number of pre–and post–migration traumatic events
							Multivariate (adjusted for age, gender, year of immigration, and pre- and post-migration trauma exposure): older age, higher number of pre and post–migration traumatic events
Matheson et al. (2008)	90 Somali (Canada)	≥9 (90 % sample) of residence	non–random (−)	IES-R	IES-R: Reported as previously validated in other non-refugee cultures (data not reported).	Depression 22.5 %	DEPRESSION
				-PTSD		PTSD = 22.2 %	Univariate: higher number of traumatic events, assault from a stranger or familiar other, coping strategies involving engagement with emotions and avoidant coping efforts
				BDI			
				-Depression			
					In this study, Cronbach’s alpha =0.96.		
					BDI: Validated in other cultures (not reported in the study).		Multivariate: the relation between trauma experiences and depression was fully 9confounded by endorsement of emotion-focused coping strategies in relation to acculturation stressors
					In this study, Cronbach’s alpha = 0.90.		
							PTSD
							Univariate: higher number of traumatic events, collective trauma, threat to other, assault from a stranger or familiar other, coping strategies involving engagement with emotions and avoidant coping efforts
							Multivariate: higher number of traumatic events
Mollica et al. (1998)	993 Cambodian (refugee camp on Thailand–Cambodia border)	≥5 of residence	random (98 %)	HSCL–25	HTQ and HSCL-25: Reported as validated in this culture (data not reported).	Depression 55 %	DEPRESSION, PTSD, PTSD SUB–SCALES (except avoidance, which had no dose–effect relationship), ANXIETY
				-Depression		PTSD = 14.7 %	
				-Anxiety			
				HTQ			
				-PTSD			
							Multivariate (covariates: gender, age, marital status, education, trauma exposure, and one of the symptom scale or sub-scales): dose–response relationship between cumulative trauma and symptoms – recent trauma had a more potent effect except for emotional numbing (roughly equally 'potent')
Nicholson (1997)	447 Southeast Asian (Vietnamese, Cambodians, Laotians and Hmong) (USA)	9.2 of residence	non–random (−)	HTQ	HTQ: Reported as validated in this culture (data not reported).	Depression = 40 %	PTSD, ANXIETY, DEPRESSION
				-PTSD		PTSD = 14 %,	
				HSCL–25		Anxiety = 35 %	Multivariate: degree of current stress was the strongest predictor, self–perceived poor health status, greater number of experienced traumatic events (in particular for PTSD), while greater number of witnessed events and rural background (confounded through current stress), and female gender (confounded through lower income) had indirect effects on all mental health. In addition, low income and being unmarried predicted depression; greater number of witnessed events predicted PTSD; and female gender predicted anxiety
				-Depression			
				-Anxiety	In this study, Cronbach’s alpha =0.95.		
					HSCL-25: Reported as validated in this culture (data not reported).		
					In this study, Cronbach’s alpha for depression = 0.89 and anxiety = 0.89.		
Onyut et al. (2009)	1422 Somalis and Rwandans (refugee camp in Uganda)	≥9 (80 % sample) of residence	random (<90 %)	PDS	PDS: Reported as previously validated in other non-refugee cultures (data not reported).	PTSD = 37.8 %	DEPRESSION
				-PTSD			Univariate: male gender (only for Rwandese), being Somali, higher number of traumatic events, functioning deficits, physical health deficits
				HSCL-25			
				-Depression			
				-Anxiety			
					In this study, sensitivity = 0.86 and specificity = 0.88		
							PTSD
							Univariate: male gender (only for Rwandese), being Somali, higher number of traumatic events, functioning deficits, physical health deficits
					HSCL-25: Validated in other refugee cultures (not reported in the study).		
					In this study, for depression subscale sensitivity = 0.67 and		ANXIETY
							Univariate: female gender (only for Somali), being Somali, higher number of traumatic events, functioning deficits, physical health deficits
					specificity = 0.73.		
Sabin et al. (2003)	170 Guatemalan (refugee camp in Mexico)	20 of residence	random (93 %)	HSCL–25	HSCL-25 and HTQ: Reported as previously validated in other refugee cultures (data not reported).	Depression = 38.8 %	DEPRESSION
				-Depression			
						PTSD = 11.8 %	Univariate: female, widowed, witnessing disappearance of others, torture, mutilation, higher number of traumatic events
				-Anxiety		Anxiety = 54.4 %	
				HTQ			
				-PTSD			
					In this study, HTQ Cronbach’s alpha =0.87; HSCL-25 Cronbach’s alpha =0.95.		
							Multivariate: female, widowed, witnessing disappearance of others, higher number of traumatic events
							PTSD
							Univariate: older age, being close to death, witnessed assassination or massacre, disappearance of others, larger household size, lived in 3 or more camps, not having experienced lack of food
							Multivariate: disappearance of others, being close to death, larger household size, and not having experienced lack of food
							ANXIETY
							Univariate: older age, higher number of traumatic events, sexual abuse or rape, witnessing massacre, witnessing disappearance of others, torture
							Multivariate: witnessing a massacre, higher number of traumatic events
Schweitzer et al. (2006)	63 Sudanese (Australia)	9 since leaving home country (2 of residence)	non–random (−)	HSCL-37	HSCL-25 and HTQ: Reported as previously validated in other refugee cultures (data not reported).	Depression =16 %	DEPRESSION
				-Depression		PTSD = 13 %	Univariate: female, higher number of traumas experienced by family, longer time in transit, family separation, less of ethnic community support
				-Anxiety			
				HTQ			
				-PTSD			
					In this study, HTQ Cronbach’s alpha =0.87; HSCL-25 Cronbach’s alpha for depression = 0.89 and for anxiety = 0.82		
							Multivariate: female, higher number of trauma experienced by family, longer time in transit, longer residence, family separation, unemployment
							PTSD
							Univariate: female, higher number of trauma experienced by the individual, higher number of trauma experienced by family, less of ethnic community support, more post-migration living difficulties
							Multivariate: female, higher number of trauma experienced by the individual, higher number of trauma experienced by family, less of ethnic community support
							ANXIETY
							Univariate: female, higher number of trauma experienced by the individual, number of trauma experienced by family, less of ethnic
							community support, more post-migration living difficulties
							Multivariate: female, higher number of trauma experienced by family, longer residence, less of ethnic community support, unemployment, more post-migration living difficulties
Steel et al. (2002)	1161 Vietnamese (Australia)	11.4 of residence	random (82 %)	CIDI, PVPS	PVPS: Developed in this culture (data not reported)	Depression =3 %	ANXIETY DISORDERS
						PTSD = 4 %	Univariate: females
				-Depression		GAD = 0.7 %	
						Social Phobia = 0.3 %	
				-PTSD			
				-GAD		Panic disorder = 0.6 %	
				-Social phobia			
						OCD = 0.5 %	
				-Panic		PVPS: similar results	
				disorder			
				-OCD			
Stige & Sveaass (2010)	142 Sri Lankan Tamils & Aceh from Indonesia (Norway)	6.7 of residence	combination of random (10 %) and non-random	PTSS	Validated in other non-refugee cultures (not reported in the study).	PTSD = 75.4 %	Not reported
				-PTSD			
					In this study, Cronbach’s alpha = 0.98		
von Lersner et al. (2008)	100 refugees from former Yugoslavia (85 %), Turkey (8 %) and Iraq (5 %) (Germany)	10.8 of residence	non–random (−)	MINI	MINI: Reported as previously validated in other non-refugee cultures (data not reported).	Depression = 42.0 %	Not reported
				-Depression			
						PTSD = 44.2 %	
				-Anxiety		GAD = 2 %	
				-Social phobia		Social phobia = 9.6 %	
					PDS: Validated in other refugee cultures (not reported in the study).	Panic disorder = 8 %	
				-Panic disorder			
						Agoraphobia = 9 %	
				-Agoraphobia		OCD = 0 %	
				PDS			
				-PTSD			
Westermeyer (1988)	97 Hmong from Laos (USA)	7–9 since leaving home country (6–8 of residence)	random (95 %; 96 % at the follow–up assessment)	NIMH DIS	Validated in other non-refugee cultures (not reported in the study).	Axis 1 disorder = 44 %	Not reported
				-Depression			
						Depression = 6.2 %	
				-GAD		GAD = 1 %	
				-OCD		OCD = 0.4 %	

Scales: *BDI* Beck Depression Inventory, *CES*–*D* Center for Epidemiologic Studies Depression Scale, *CIDI* Composite International Diagnostic Interview, *DICA* Diagnostic Interview for Children and Adolescents, *DICA*–*R* Diagnostic Interview for Children and Adolescents–Revised, *HOS* Health Opinion Survey, *HSCL–D* Hopkins Symptom Checklist–Depression Scale, *HSCL–25* Hopkins Symptoms Checklist–25, *HTQ* Harvard Trauma Questionnaire, *IES–R* Impact of Event Scale–Revised, *MHI* Mental Health Inventory, *MINI* The Mini International Neuropsychiatric Interview, *PVPS* Phan Vietnamese Psychiatric Scale

Thirteen studies were determined to be of higher quality [15, 28, 30, 47, 48, 51–58] and the remaining 16 of lower methodological quality. Just over half of the studies applied probability sampling methods, although the sampling frame from which the samples were drawn varied: three drew their sample randomly from national refugee census data [47, 53, 54], three used multi-cluster random selection [15, 55, 58], three conducted a household survey in refugee camps [56, 57, 59] with one study also employing a single-cluster random selection [56], two studies used a community panel database [13, 48], one used health screening records [60], one drew its sample from a telephone directory book [51], one included the entire refugee sample in a given geographical area [28] and for one study the sampling frame was unclear [61]. Of these, only three studies reported response rates below 60 % [47, 48, 61] and two did not report response rate [54, 59]. Other studies drew their sample using non-random sampling methods or a combination of random and non-random methods.

In six studies diagnoses were made using structured clinical interviews [15, 28, 52, 58, 60, 62], but in most studies diagnoses were based solely on self-report questionnaires, with the Hopkins Symptom Checklist-25 (HSCL-25) [70] being the most frequently used for the assessment of depression or unspecified anxiety and the Harvard Trauma Questionnaire (HTQ) [71] for PTSD. Other anxiety disorders were assessed with a structured clinical interview, including the National Institute of Mental Health Diagnostic Interview Schedule (NIMH DIS) [72], the CIDI [27], and the Mini International Neuropsychiatric Interview (MINI) [73].

Of the 16 instruments used to assess mental health, only three instruments (HTQ; PVPS; Unnamed instrument by Beiser & Hou, 2001) were developed specifically for a refugee population. Although most of the instruments had been previously tested and validated among various cultures, only ten studies used instruments that had been previously validated specifically in the refugee culture being observed. As displayed in Table 2, 62 % (*N* = 18) of the studies reported estimates of reliability and/or validity of the used instruments, including internal consistency (Cronbach’s alpha), test-retest reliability, specificity, and sensitivity. Most of the described reliability and/or validity data were based on the instruments performance in the current study (*N* = 17), while only five studies reported reliability and/or validity data from both a prior and the current study. However, 38 % (*N* = 11) of the studies reported no reliability or validity data and three studies made no mention of the reliability or validity of the used instruments.

In terms of language used for the assessment, in all but one study [69] the interview was conducted in a native language. Of these, in 21 studies interviews were conducted with a bilingual researcher (unless a postal questionnaire) using translated instruments; in one study Western psychologists conducted interviews using translated instruments with the help of trained local interviewers and translators [59]; one study used translated instruments for assessing mental health but not for other data collection, which was conducted with the help of a translator [14]; one used translated instruments for one cultural subsample but not for another, for which interpreters were available throughout the interview [67]. In five studies the instruments were not translated but the interviews were conducted in a native language through either an interpreter [13, 62] or a bilingual researcher who translated the questions to the participants in vivo (e.g. Mayan language is not a written language and bilingual interviewers translated the questions, in vivo, from the questionnaires written in Spanish) [28, 57, 68]). Three studies offered for the interview to be done in either a native or host language [11, 66, 68], although it is not clear what percentage chose to be interviewed in a host language.

### Data synthesis of prevalence rates of depression and anxiety disorders

Of the 29 studies identified for the review, 27 reported on prevalence rates of depression and/or anxiety disorders and were, therefore, included in data synthesis of prevalence rates. Prevalence rates of mental disorders reported in individual studies varied widely. The overall heterogeneity between studies was very high for each disorder (*I*^*2*^ > 90 %). Therefore, it was decided that the data was not amenable to a meta-analysis. Instead, the following data syntheses and analyses focus on investigating sources of heterogeneity both quantitatively (subgroup analysis) and narratively.

### Depression

**Fig. 2:**
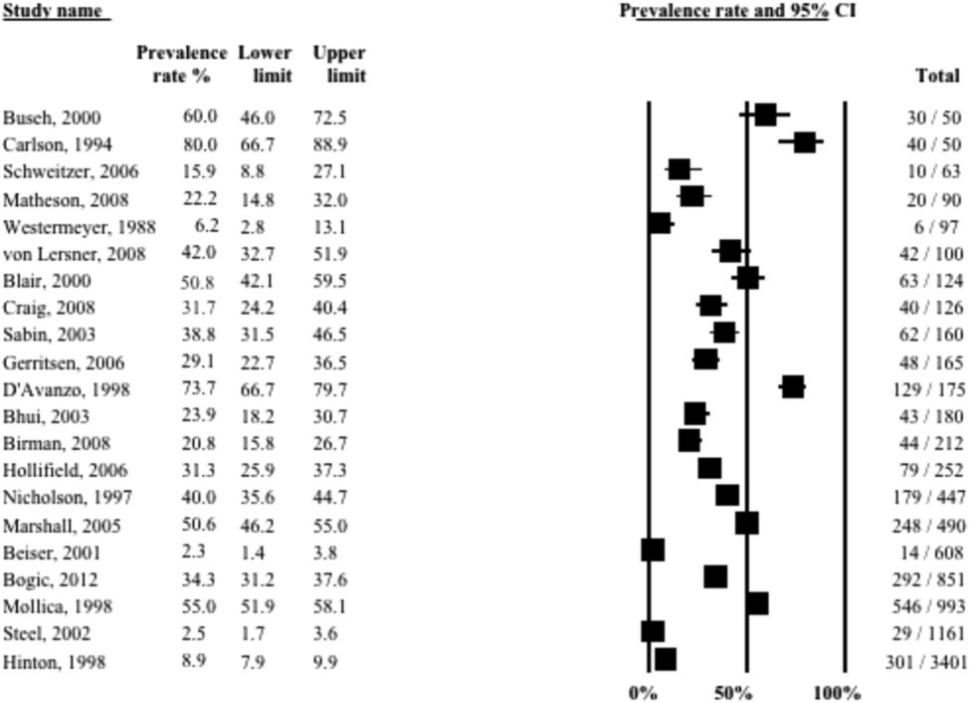
Forest plot of prevalence rates (%, with 95 % CI) of depression in long-settled war refugees in individual studies. For each study, only the name of the first author is shown

Twenty-one studies (9970 refugees; median time since displacement 9.0 years) reported on prevalence of depression. A graphical summary of individual study prevalence rates is presented in Fig. 2. As can be seen in the forest plot, there was substantial heterogeneity in depression prevalence between studies ranging from a low 2.3 % among Southeast Asian refugees in Canada [11] to a high 80 % among Cambodian refugees in the USA [13], with 76 % of the studies reporting prevalence higher than 20 %. Accordingly, the overall statistical heterogeneity between studies was very high (*Q* = 1590.0, d.f. = 20, *p* < 0.0001, *I*^*2*^ = 98.7 %).

**Fig. 3:**
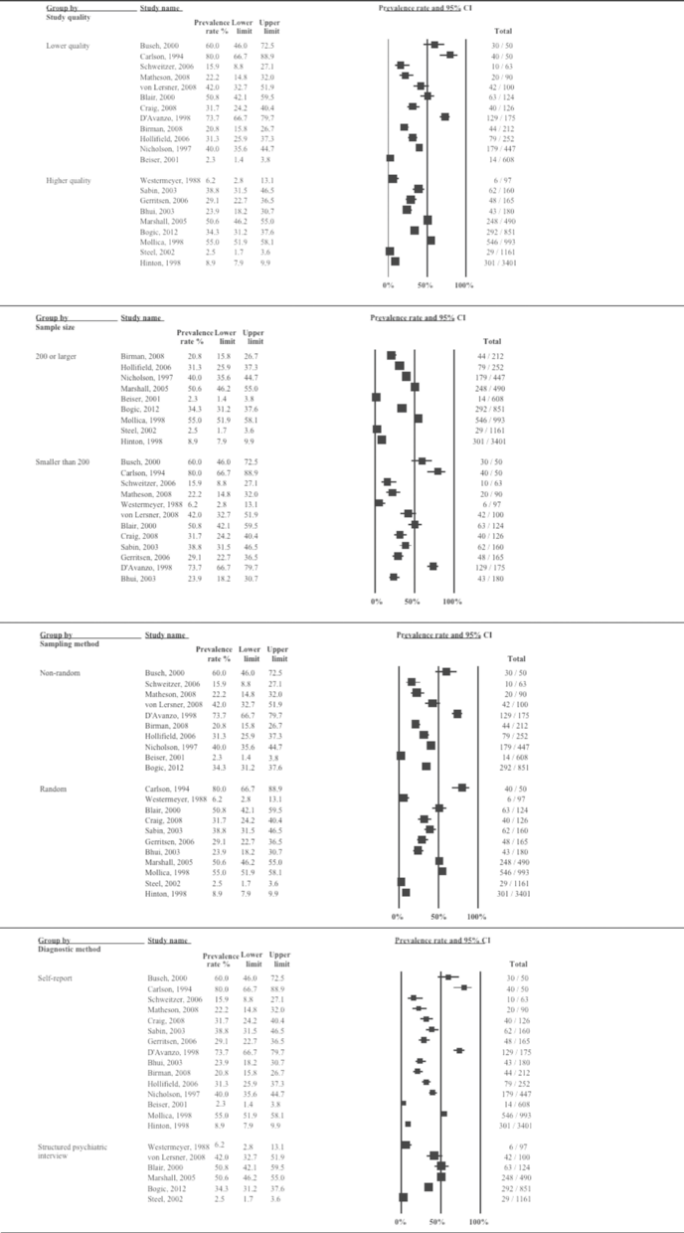
Prevalence rates (%, with 95 % CI) of depression in long-settled war refugees stratified by study characteristics: study quality, sample size, sampling and diagnostic method

**Fig. 4:**
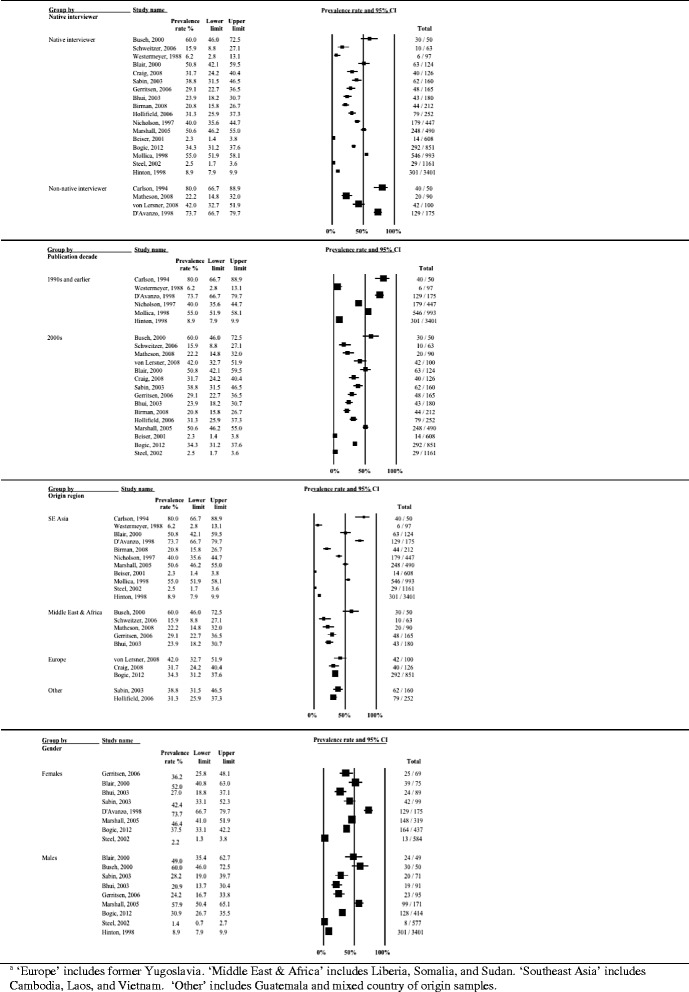
Prevalence rates (%, with 95 % CI) of depression in long-settled war refugees stratified by study and refugee characteristics: interview language, publication decade, origin region^a^, and gender. ^a^ ‘Europe’ includes former Yugoslavia. ‘Middle East & Africa’ includes Liberia, Somalia, and Sudan. ‘Southeast Asia’ includes Cambodia, Laos, and Vietnam. ‘Other’ includes Guatemala and mixed country of origin samples

**Fig. 5:**
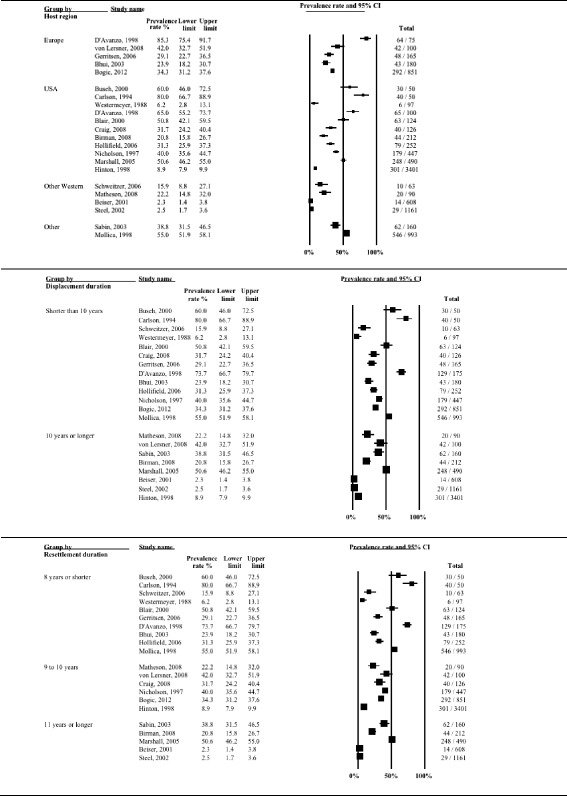
Prevalence rates (%, with 95 % CI) of depression in long-settled war refugees stratified by refugee characteristics: host region^a^, displacement and resettlement duration. ^a^‘Europe’ includes France, Germany, Italy, Netherlands, and United Kingdom. ‘Other Western’ includes Canada and Australia. ‘Other’ includes refugee camps in Mexico and on Thailand-Cambodia border

Heterogeneity between studies remained high (*I*^*2*^ > 90 %) after stratifying for clinical and methodological characteristics of the studies. The subgroup analysis indicated that heterogeneity was partly explained by displacement duration (*p* = 0.017), host region (*p* = 0.004) and the language of the interviewer (*p* = 0.035). Reported rates of depression also showed gradual decline with increasing time since resettlement in a study country, although the difference was only approaching statistical significance (*p* = 0.073). Similarly, there were differences, although only approaching statistical significance, between studies with small and large sample sizes (*p* = 0.092). Prevalence rates of depression stratified by clinical and methodological characteristics are displayed with forest plots in Figs. 3–5.

The following descriptive differences between subgroups were also observed, although they did not reach statistical significance. Whilst heterogeneity remained high in both subgroups, the group of higher methodological quality tended to report slightly lower rates of depression (2.5 to 55 %; depression prevalence <25 % in 44 % of the studies) compared to the group of lower quality (range 2.3 to 80 %; depression prevalence <25 % in 29 % of the studies) (Fig. 3). The majority of the higher quality studies were conducted in Western countries and those that were conducted in refugee camps in developing countries [55, 57] tended to report the highest prevalence in this group (38.8 to 55 %).

Heterogeneity was higher in studies with smaller sample sizes (range 6.2 to 80 %; depression prevalence >40 % in 58 % of the studies) compared to those with larger sample sizes (2.3 to 55 %; depression prevalence >40 % in 22 % of the studies) (Fig. 3). The smaller studies included studies that differed widely in the origin of the samples as well as the host region, and included studies that used non-native language interviewers. In fact, the studies with the highest prevalence were mostly conducted in non-native language (22.2 to 80 %; depression prevalence >40 % in 75 % of the studies compared to 31 % of the studies conducted in native language) (Fig. 4). The larger studies, on the other hand, mainly included samples from Southeast Asia who had been displaced for longer periods in the North America or Australia. The largest prevalence in this group was observed in a study conducted in a refugee camp in a developing country (55 %; [55]). With the exception of one outlier (50.6 %; [15]), the remaining large studies that were conducted in Western countries tended to report lower prevalence of depression (range 2.3 to 40 %).

There was very high heterogeneity in the prevalence of depression in studies conducted across different population origins (Fig. 4). With the exception of one study that assessed Liberian men [49], studies conducted with refugees originating from Middle East or Sub-Saharan Africa tended to report the lowest prevalence (16 to 29.1 %). Refugees originating from former Yugoslavia tended to have somewhat higher rates of depression (31.7 to 42 %). Southeast Asian refugees as a group showed a very high variability in the prevalence of depression (2.3 to 80 %). However, there were some notable differences in the prevalence depending on the exact country of conflict that the refugees originated from. The highest rates tended to be for Cambodian refugees (50.6 to 80 %) and substantially lower for those originating from Vietnam or Lao (2.3 to 20.8 %). This would suggest that, overall, refugees from Vietnam or Lao had the lowest rates and those from Cambodia the highest rates of depression.

In terms of the host region, studies conducted in Australia and Canada tended to report the lowest prevalence of depression (Australia, 2.5 to 15.9 %; Canada, 2.3 to 22.2 %) (Fig. 5). Studies conducted with refugees in Europe tended to report somewhat higher prevalence rates; depression was present in about a third of the sample (23.9 to 42 %). This excludes the study by D’Avanzo & Barab [14], which is an outlier with a prevalence rate of 85.3 %. This is also the only study that included only women in their sample. Again, the two studies with the highest prevalence rates were also of a lower methodological quality and both used an interpreter at the interview [14, 62].

Studies conducted in the USA indicated generally the highest rates of depression in refugees; however, there was very high variability in the rates reported (6.2 to 80 %). This group included mostly studies of lower methodological quality. Contrary to the finding of the trend for the highest rates in the USA, the study by D’Avanzo & Barab [14] indicated that Cambodian refugee women residing in France were significantly more likely to show symptoms indicative of depression than Cambodian refugee women residing in the USA (85.3 % vs. 65 %, respectively). The authors suggested social factors of the new environment as an explanation, rather than differences in pre-migration experiences. However, none of the potential confounders (age, education, traumatic history, employment) were adjusted for in the analysis. Studies conducted in refugee camps reported rates from 38.8 to 55 %.

Only two studies included in the review assessed the prevalence of depression longitudinally [11, 28]. Both studies followed Southeast Asian refugees during their 10-year resettlement period in North America and found that depression level scores improved substantially during this period. Although initial levels of depression were low (6.5 %), Besier and Hou [11] recorded further reduction in depressive symptoms over time with the rates falling to below that of the host population by the end of the 10-year period (2.3 %). Data from the cross-sectional studies that were taken at different time points in the refugee displacement also suggest that longer-displaced refugees (10 years or longer in resettlement) tended to report lower rates of depression (2.3 to 50.6 %; in 70 % of the studies prevalence below 25 %) compared to those with shorter displacement duration (6.2 to 80 %; 21 % of the studies below 25 %) (Fig. 5).

### Anxiety disorders

Prevalence rates of anxiety disorders were reported in 24 studies (9401 refugees; median time since displacement 9.0 years). Of these, nine studies used a screening questionnaire (of which eight used the HSCL-25 anxiety subscale) that did not specify the type of anxiety disorder [13, 14, 47, 48, 57, 61, 63–65]. Of those that assessed a specific anxiety disorder, 20 studies reported on prevalence of PTSD, five reported on Generalized Anxiety Disorder (GAD) [28, 52, 58, 60, 62], four each on Panic Disorder [52, 58, 60, 62], Social Phobia [52, 58, 60, 62], and Obsessive-Compulsive Disorder (OCD) [28, 52, 58, 62], and two reported on prevalence of Agoraphobia [52, 62]. The findings of the studies are reported separately for each group of disorders i.e. unspecified anxiety, PTSD, GAD, Panic Disorder, Social Phobia, OCD, and Agoraphobia.

### Unspecified anxiety

**Fig. 6:**
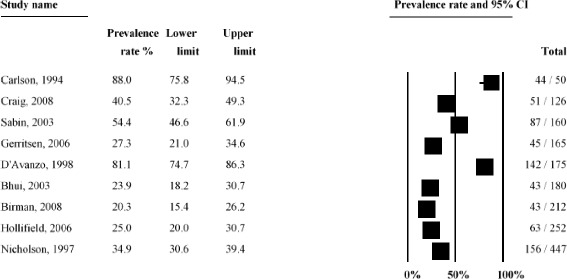
Forest plot of prevalence rates (%, with 95 % CI) of unspecified anxiety in long-settled war refugees in individual studies

A graphical summary of the study prevalence rates for unspecified anxiety is presented in Fig. 6. There was substantial heterogeneity in anxiety prevalence between studies ranging from a low 20.3 % [63] to a high 88 % [13] among Southeast Asian refugees in the USA. Accordingly, the overall statistical heterogeneity between studies was very high (*Q* = 219.8, d.f. = 8, *p* < 0.0001, *I*^*2*^ = 96.4 %).

**Fig. 7:**
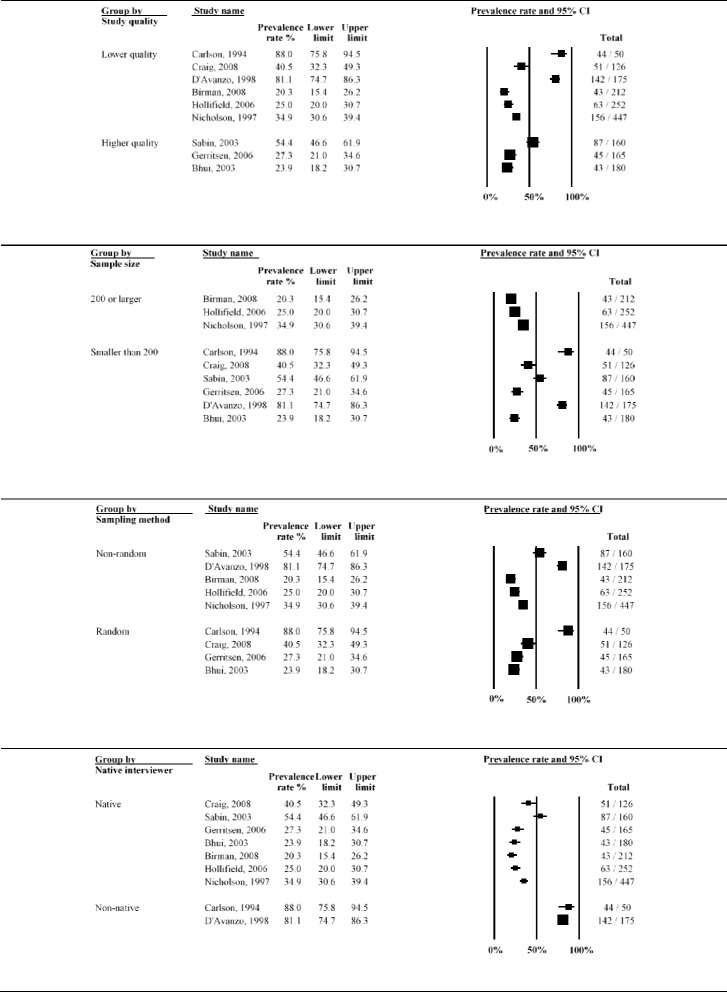
Prevalence rates (%, with 95 % CI) of unspecified anxiety in long-settled war refugees stratified by study characteristics: study quality, sample size, sampling method, and interview language

**Fig. 8:**
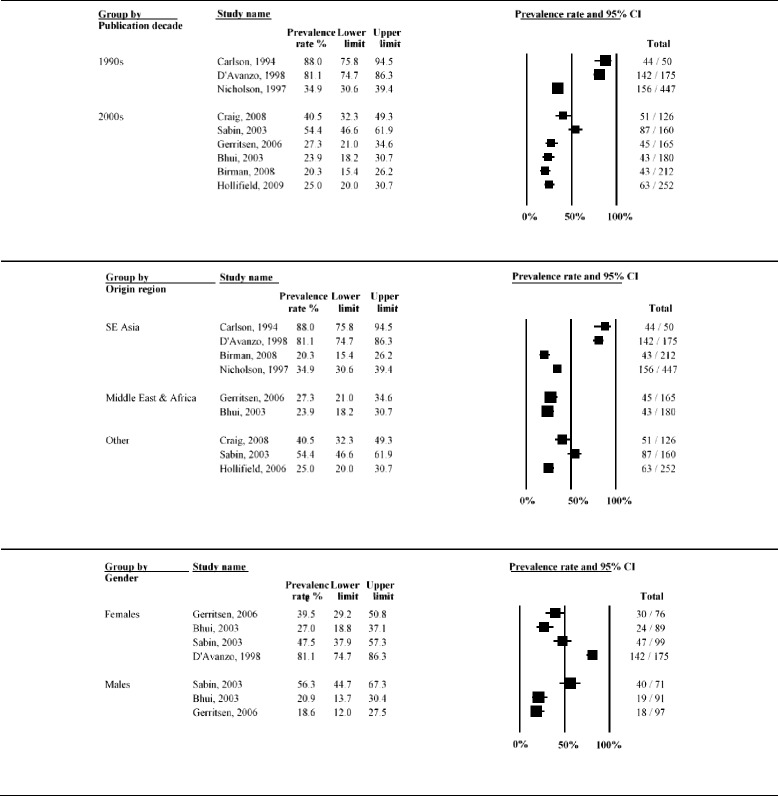
Prevalence rates (%, with 95 % CI) of unspecified anxiety in long-settled war refugees stratified by study and refugee characteristics: publication decade, origin region^a^ and gender. ^a^ ‘Southeast Asia’ includes Cambodia, Laos, Sri Lanka, and Vietnam. ‘Middle East & Africa’ includes Afghanistan, Iran, Somalia, and Sudan. ‘Other’ includes Guatemala, Bosnia, and mixed country of origin samples

**Fig. 9:**
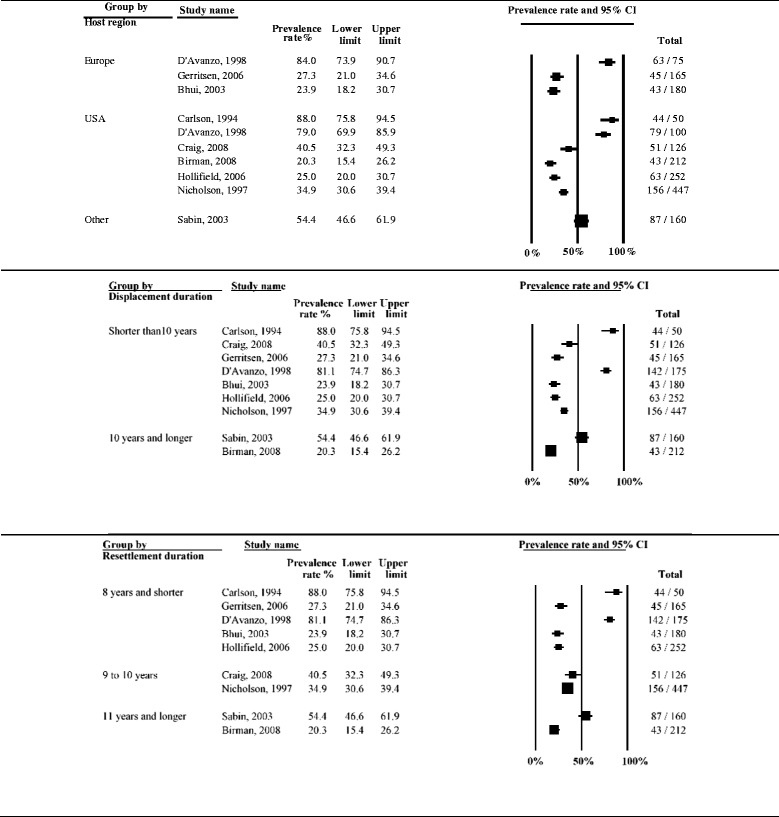
Prevalence rates (%, with 95 % CI) of unspecified anxiety in long-settled war refugees stratified by refugee characteristics: host region^a^, displacement and resettlement duration. ^a^ ‘Europe’ includes France, The Netherlands, and United Kingdom. ‘Other’ includes Mexico

Heterogeneity in the prevalence of unspecified anxiety remained very high (*I*^*2*^ > 88 %) even after the studies were stratified by clinical and methodological characteristics. Prevalence rates stratified by study and sample characteristics are shown in Figs. 7–9.

Subgroup analysis indicated that the between-study heterogeneity was partly explained by differences between studies with small and large sample sizes (*p* = 0.02) and studies in which interviews were done by native and non-native speakers (*p* < 0.001), although the latter finding is limited as only two studies used non-native speaking interviewers. There was also a marginally significant statistical difference between studies that were published before 2000 and those published after (*p* = 0.06).

The following descriptive differences between subgroups were also observed, although they did not reach statistical significance. Heterogeneity in prevalence was somewhat lower in studies of higher methodological quality (range 23.9 to 54.4 %) compared to the studies of lower quality (20.3 to 88 %) (Fig. 7). Two of the higher quality studies were conducted in Europe with refugees coming from the Middle East or Sub-Saharan Africa and both reported similar rates (23.9 and 27.3 %), whereas the third one was conducted with Guatemalan refugees in a refugee camp in Mexico and reported substantially higher rates (54.4 %). Studies of lower methodological quality included refugees of various origin and host regions. In this group, the highest prevalence rates were reported in studies conducted with Cambodian refugees (81.1 to 88 %) and the lowest in Vietnamese refugees (20.3 %). Smaller studies and those conducted in a non-native language reported the highest rates of unspecified anxiety (Fig. 7).

There was some evidence for lower rates of unspecified anxiety to be reported for males (18.6 to 56.3 %) compared to females (27 to 81.1 %) (Fig. 8). With the exception of the study by Sabin et al. [57], the studies that reported on prevalence in males and females separately reported higher rates of unspecified anxiety in females.

In terms of the host country, the highest rates were reported in studies conducted in the USA; however the heterogeneity between these studies was substantial (20.3 to 88 %) (Fig. 9). These studies were also all rated as of lower methodological quality. With the exception of a study of Cambodian females [14], studies conducted in Europe tended to report the lowest rates (23.9 to 27.3 %).

### Posttraumatic Stress Disorder (PTSD)

**Fig. 10:**
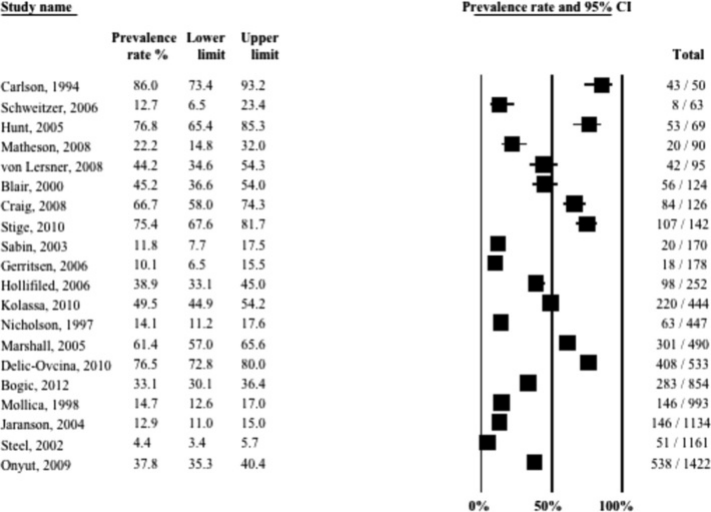
Forest plot of prevalence rates (%, with 95 % CI) of PTSD in long-settled war refugees in individual studies. For each study, only the name of the first author is shown

Twenty studies (8837 refugees; median time since displacement 9.0 years) reported on PTSD prevalence. A graphical summary of study prevalence rates is presented in Fig. 10. There was substantial heterogeneity in prevalence rates of PTSD across studies, with prevalence ranging from 4.4 % [58] to 86 % [13]. Over two thirds of studies reported PTSD prevalence higher than 20 %. The overall statistical heterogeneity between studies was very high (*Q* = 1546.9, d.f. = 19, *P* < 0.0001, *I*^*2*^ = 98.8 %).

**Fig. 11:**
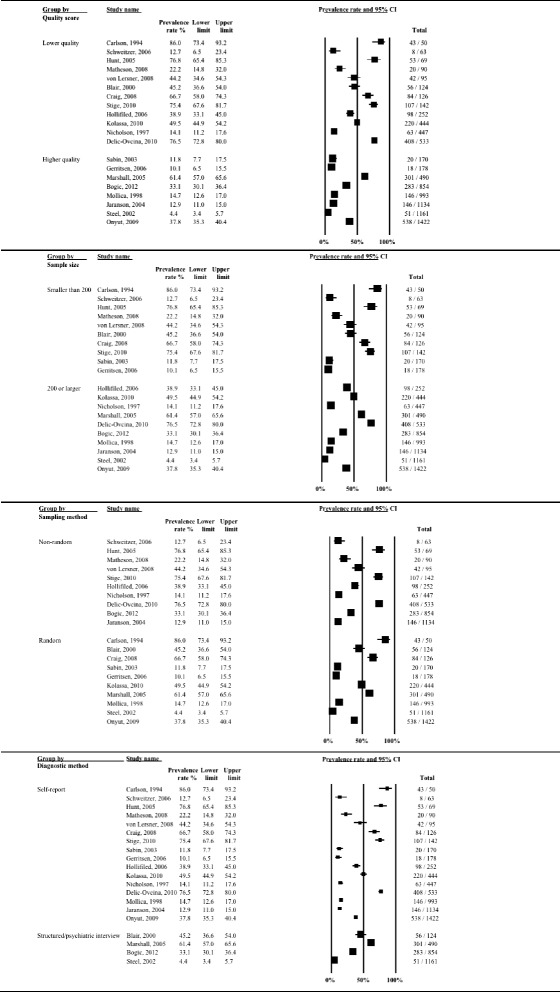
Prevalence rates (%, with 95 % CI) of PTSD in long-settled war refugees stratified by study characteristics: study quality, sample size, sampling and diagnostic method

**Fig. 12:**
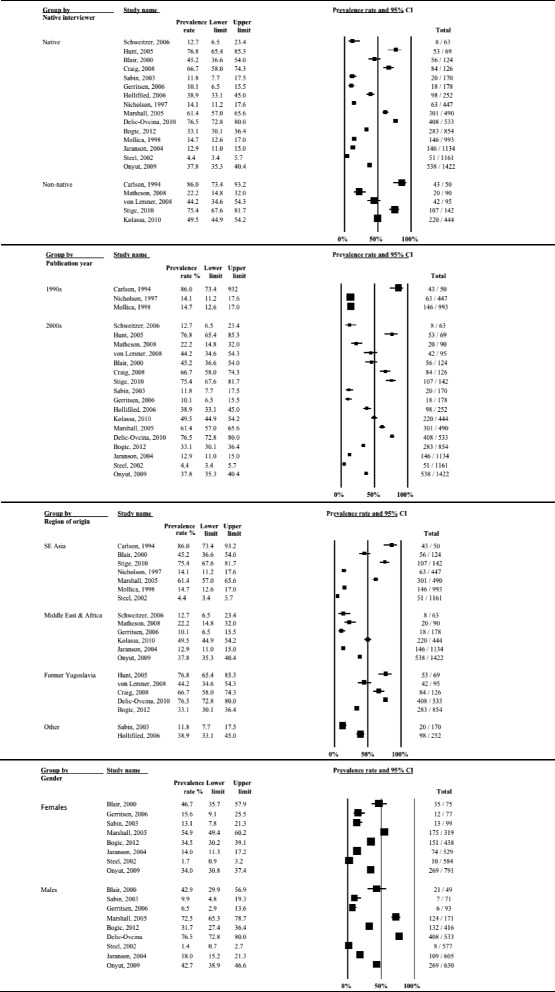
Prevalence rates (%, with 95 % CI) of PTSD in long-settled war refugees stratified by study and refugee characteristics: interview language, publication decade, region of origin, and gender. ^a ‘^Europe’ includes former Yugoslavia. ‘Middle East & Africa’ includes Afghanistan, Ethiopia, Iran, Rwanda, and Somalia. ‘Southeast Asia’ includes Cambodia, Indonesia, Laos, Sri Lanka, and Vietnam. ‘Other’ includes Guatemala and mixed country of origin samples

**Fig. 13:**
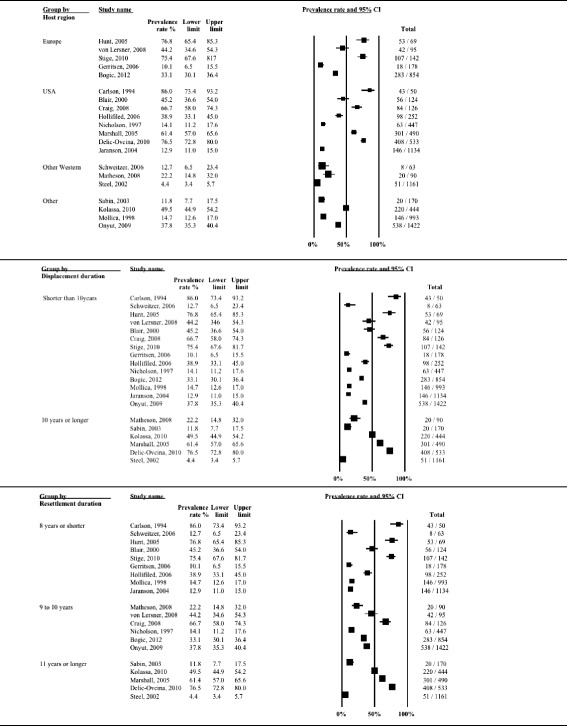
Prevalence rates (%, with 95 % CI) of PTSD in long-settled war refugees stratified by refugee characteristics: host region, displacement and resettlement duration,. ^a^ ‘Europe’ includes Germany, The Netherlands, Norway, and United Kingdom. ‘Other Western’ includes Australia and Canada. ‘Other’ includes refugee camps in Mexico, Uganda and on Thailand-Cambodia border

Subgroup analysis indicated that this heterogeneity was partly due to the differences between studies of higher and lower quality (*p* = 0.002) and between studies conducted in the USA, Europe, other Western regions and those done elsewhere (*p* = 0.043). There was a marginally significant statistical difference between studies depending on whether interviews were conducted by native speaker or not (*p* = 0.071) and between studies with samples originating from Europe (former Yugoslavia), SE Asia, Middle East and Africa and other regions (*p* = 0.051). The heterogeneity remained very high even after the studies were stratified by methodological and clinical characteristics (*I*^*2*^ > 90 %). PTSD prevalence rates stratified by study and sample characteristics are displayed with forest plots in Fig. 11–13.

As noted above, prevalence of PTSD was the highest (12.7 to 86 %) in studies of lower methodological quality, with more than two thirds reporting rates higher than 40 % (Fig. 11). Five studies with the highest rates of PTSD (66.7 to 86 %) were all of lower methodological quality. Lower quality studies that used postal questionnaires [66, 67] or non-native interviewer [13, 67] tended to report the highest PTSD rates. Studies of higher quality tended to report lower and less variable rates (4.4 to 61.4 %). For example, the three studies with the lowest rates of PTSD (4.4 to 11.8 %) were all of a higher quality. With the exception of Marshall et al. [15], reported rates were below 40 % and over two thirds reported rates below 15 %.

In terms of rates of PTSD by region of origin, there was a high variation (Fig. 12). The lowest rates tended to be reported for refugees originating from Sub-Saharan Africa (12.7 to 49.5 %), whilst the highest were reported for refugees from Europe (mainly from former Yugoslavia) (33.1 to 76.8 %) and for Southeast Asian refugees (14.1 to 86 %), particularly for refugees from Cambodia resettled in the USA (45.2 to 86 %).

Studies conducted in Australia tended to report the lowest rates of PTSD (4.4 to 12.7 %), followed by a study from Canada (22.2 %), and the studies conducted in refugee camps (11.8 to 49.5 %) (Fig. 13). Compared to studies conducted in refugee camps in Mexico (11.8 %) and Thailand (14.7 %), studies conducted in refugee camps in Sub-Saharan Africa (Uganda) reported higher rates of PTSD (37.8 to 49.5 %). Prevalence of PTSD reported in studies conducted in the USA were highly variable (12.9 to 86 %), although majority (63 %) of the studies reported rates above 40 % and these high rate studies included refugees from former Yugoslavia and Cambodia.

### Other anxiety disorders

Five studies reported on GAD [28, 52, 58, 60, 62], with rates ranging from 0.7 to 13.7 % (*Q* = 64.1, d.f. = 4, *p* < 0.0001, *I*^*2*^ = 93.8 %). Rates of Panic Disorder ranged from 0.6 to 10 % (*Q* = 71.2, d.f. = 3, *p* < 0.0001, *I*^*2*^ = 95.8 %) [52, 58, 60, 62], Social Phobia from 0.3 to 27.4 % (*Q* = 108.0, d.f. = 3, *p* < 0.0001, *I*^*2*^ = 97.2 %) [52, 58, 60, 62], OCD from 0 to 5 % (*Q* = 28.8, d.f. = 3, *p* = 0.001, *I*^*2*^ = 89.6 %) [28, 52, 58, 62]. Agoraphobia rate was reported in two studies, ranging from 8.2 to 9 % (*Q* = 0.1, d.f. = 1, *p* = 0.785, *I*^*2*^ = 0 %) [52, 62]. Overall, the lowest rates for these disorders tended to be reported by a higher quality study conducted with Vietnamese refugees in Australia [58] and the highest by a lower quality study of Cambodian refugees in the USA [60]. A subgroup analysis was not performed for the remaining anxiety disorders because of the small number of the studies.

### Comorbidity of mental disorders

Three studies provided some data on comorbidity of mental disorders. 68.4 % of those diagnosed with PTSD also had a diagnosis of depression [15, 52, 60]; 43.2 % of those who had GAD also had PTSD, whilst 50.7 % also had Major Depression [52]; and 22.4 % of those who had PTSD also had Social Phobia [52, 60].

### Publication bias

Based on the Begg-Mazumdar rank correlation test and Egger’s linear regression method, there was no evidence of publication bias for depression (Begg-Mazumdar *p* = 0.132, Egger’s *p* = 0.484) or PTSD (Begg-Mazumdar *p* = 0.487, Egger’s *p* = 0.481). The results for unspecified anxiety disorder studies were less clear as Begg-Mazumdar rank correlation test was marginally statistically significant, whereas Egger’s linear regression test was statistically non-significant (Begg-Mazumdar *p* = 0.06, Egger’s *p* = 0.133).

### Factors associated with mental disorders - a narrative review

Twenty-five studies reported on risk factors for depression, PTSD, and/or unspecified anxiety disorder, using univariate analysis [13, 14, 49, 50, 56, 58, 60, 61]), multivariate analysis [53, 55, 59, 65], or both [11, 15, 30, 47, 48, 51, 52, 54, 57, 63, 64, 68, 69]. Eleven risk factors were studied by either univariate or multivariate analysis in at least three studies for one of the three outcomes. These factors can be classified into three major categories: demographics, war-related, and post-migration factors. The findings are presented in Table 3.

**Table 3:** Summary of risk factors for depression, PTSD, or unspecified anxiety from univariate and multivariate analysis of studies included in the review^a,b^

	Depression		PTSD		Unspecified anxiety	
Factor	Univariate	Multivariate	Univariate	Multivariate	Univariate	Multivariate
Demographics						
older age	+ (4/11; 36 %)	+ (2/9; 22 %)	+ (5/8; 63 %)	+ (2/6; 33 %)	+ (2/5; 40 %)	+ (1/5; 20 %)
female gender	+ (3/9; 33 %)	+ (4/9; 44 %)	+ (1/6; 17 %)/–(1/6; 17 %)	+ (2/6; 33 %)/–(1/6; 17 %)	+ (2/7; 29 %)	+ (5/7; 71 %)
lower education	+ (3/5; 60 %)	+ (1/7; 14 %)	+ (3/4; 75 %)	+ (1/4; 25 %)	0 (0/1; 0 %)	0 (0/3; 0 %)
War context						
number of war traumatic events	+ (7/9; 78 %)	+ (9/12; 73 %)	+ (9/10; 90 %)	+ (9/9; 100 %)	+ (5/5; 100 %)	+ (8/9; 89 %)
combat experience	+ (1/3; 33 %)	+ (1/3; 33 %)/–(1/3; 33 %)	NA	NA	NA	NA
Post–migratin context						
longer duration in exile	– (2/10; 20 %)	+ (2/7; 29 %)	0 (0/7; 0 %)	0 (0/4; 0 %)	– (1/3; 33 %)	+ (2/3; 67 %)
post-migration stress	+ (3/4; 75 %)	+ (3/3; 100 %)	+ (3/3; 100 %)	+ (3/4; 75 %)	+ (1/1; 100 %)	+ (3/3; 100 %)
unemploymet	+ (5/6; 83 %)	+ (5/6; 83 %)	+ (2/4; 50 %)	0 (0/3; 0 %)	+ (1/2; 50 %)	+ (2/3; 67 %)
low income	+ (3/4; 75 %)	+ (3/4; 75 %)	NA	NA	NA	NA
poor host language proficiency	+ (3/4; 75 %)	+ (3/5; 60 %)	NA	NA	NA	NA
lack of social support	+ (2/2; 100 %)	+ (3/3; 100 %)	NA	NA	NA	NA
unmarrid	+ (2/4; 50 %)	+ (2/4; 50 %)	+ (1/3; 33 %)	0 (0/4; 0 %)	+ (1/1; 100 %)	0 (0/3; 0 %)

^a^‘+’ and ‘– ‘signs indicate whether a factor was positively or negatively associated with a disorder, whilst ‘0’ indicates the factor had no effect of either type; NA indicates the factor was not studied in three or more studies. Each of the risk factors shown was examined in three or more studies at least for one of the three review outcomes

^b^number of studies with +/− /0 association/ out of total number of studies that tested a factor

In summary, the majority of studies reported no association between demographic factors (age, gender, and education) and mental disorders. There was some evidence suggesting females to be at a greater risk of having unspecified anxiety disorder (5 out of 8 studies, 63 %; [47, 54, 63, 65, 68]), but not PTSD. The few studies that did report a significant association in univariate analyses between age and education and mental disorders (in particular, depression and PTSD), all indicate older age [15, 50–52, 57, 61] and lower education [50–52, 61] to be risk factors for mental disorders. However, these associations mostly disappeared once other potential confounders were adjusted for in multivariate analyses.

With respect to war-related factors, a higher number of traumatic experiences was the most common factor consistently found to be positively associated with mental disorders (≥75 % of the studies; [13, 15, 30, 47, 48, 52, 54–57, 59, 60, 63–65, 68, 69]). A longitudinal study [29] followed a small subsample of Oromo and Somali refugees in the USA (39 % of the original sample; [30]) and found that, in addition to PTSD symptom levels at the baseline assessment, pre-migration trauma was the strongest predictor of PTSD symptoms at the 3-months to 3.5 years follow-up assessment. Impact of combat was evaluated in only a few studies and was found to be related to mental disorders only in single studies [48, 51, 52].

Among the post-migration factors, higher number of post-migration stressors or higher level of distress experienced due to these stressors were positively associated with mental disorders (75–100 % of studies; [47, 49, 52, 60, 65, 68]). When considered together, poor socio-economic factors after migration (unemployment, low income, poor host language proficiency, and lack of social support) were positively associated with depression (60–100 % of studies; [11, 15, 47, 51, 52, 54, 60, 63, 65, 68]). A positive univariate association was also found between unemployment and unspecified anxiety disorder (67 % of studies; [48, 54, 68]), whilst multivariate analysis indicated an indeterminate association between the two (two out of four studies, 50 %; [54, 68]). The relationship between the remaining socio-economic factors and PTSD and unspecified anxiety disorder was assessed in fewer than three studies, rendering synthesis of results difficult. Nevertheless, these incidental findings suggest also a positive univariate association between these factors and PTSD and unspecified anxiety disorder [15, 47, 48, 50, 52, 63], but these associations mostly disappeared once other potential confounders were adjusted for in multivariate analyses, rendering the relationships either as with no association or indeterminate.

Findings on the association between duration in exile and mental disorders suggest no association between the two, with only two studies reporting association between longer duration in exile and depression and unspecified anxiety [54, 68]. Overall, studies reported no association between marital status and PTSD and unspecified anxiety, whilst the relationship with depression was indeterminate (2 out of 4 studies, 50 %; [57, 65]).

## Discussion

The current study systematically reviewed studies assessing mental disorders of long-settled war-refugees worldwide, applied a subgroup analysis to understand the reasons for prevalence variability, and narratively reviewed pre-migration and post-migration factors associated with mental health of long-settled war refugees. The review identified 29 studies on long-term mental health with a total of 16,010 war-affected refugees. The findings indicate 1) generally high prevalence rates of depression, PTSD and other anxiety disorders among refugees 5 years or longer after displacement, with prevalence estimates typically in the range of 20 % and above; 2) a large variability of prevalence rates between studies, with both clinical and methodological factors contributing substantially to the observed variability; and 3) a number of shared and unique risk factors for mental disorders. Specifically, higher exposure to traumatic experiences and post-migration stress were the most common factors consistently associated with higher rates of mental disorders. Additionally, poor post-migration socio-economic situation (unemployment, low income, poor host language proficiency, and lack of social support) was particularly associated with depression, whilst female gender was associated with unspecified anxiety, but not with PTSD. Other socio-demographic characteristics of the sample appeared to be poor predictors of long-term mental health.

The findings might have several implications. First, they indicate that the risk of having a serious mental disorder is substantially higher in war refugees than in the general population, even several years after refugee resettlement. With the exception of a few studies [11, 28, 58], the refugee studies reported prevalence rates of mental disorders that are substantially higher than those reported in studies with general non-war affected population. For example, compared with the general Western adult population [74–76], the review findings of higher quality studies indicate that refugees may be roughly up to 14 times more likely to have depression and 15 times more likely to have PTSD.

Second, the current review identified a considerable heterogeneity of prevalence rates across the studies. Prevalence rates of depression have ranged from 2.3 % [11] to 80 % [13], rates of PTSD from 4.4 % [58] to 86 % [13]. Other anxiety disorders were less often assessed in the studies. The rates of unspecified anxiety, an outcome that largely overlaps with PTSD and other anxiety disorders, have ranged from 20.3 % [63] to 88 % [13]. Overall, these studies have shown up to 40-fold difference in prevalence rates, reflecting high degree of statistical heterogeneity (*I*^*2*^ > 96 %). Such high level of heterogeneity has also been reported in previous systematic reviews and meta-analyses [8, 9].

Some degree of between-study heterogeneity is expected given the clinical and methodological differences between the studies, both in terms of participant and study characteristics. It was decided to include studies on different refugee populations in different resettlement countries at different time points of the resettlement process. These studies had notable differences in sampling and assessment methods. A subgroup analysis showed that observed statistical heterogeneity in prevalence rates was partly due to the overall methodological quality of the studies, with studies of higher quality generally reporting lower prevalence rates. Assessments made by native speakers (versus through an interpreter) tended to report lower prevalence rates for all disorders. Previous studies examining the issues related to the use of interpreters in medical settings indicated that communication errors may occur when eliciting information by such methods [77–79]. Within the context of refugee trauma research, the background characteristics of the interpreter, such as having a different dialect or being from a different ethnic group than the refugee being interviewed, may influence disclosure as well as translation.

These findings as well as the findings of the previous reviews [8, 9] indicated the observed between-study heterogeneity was also partly due to the clinical characteristics of the studies. Prevalence rates of mental disorders were to some extent related to both which country the refugees came from and in which country they finally resettled. Refugees from former Yugoslavia and Cambodia tended to have the highest rates of depression, PTSD and unspecified anxiety, whilst the refugees from Vietnam and Middle East and Sub-Saharan Africa had the lowest rates. Studies conducted outside Europe or the USA tended to report lower prevalence rates of mental disorders, perhaps reflecting widely different refugee selection and resettlement policies. Studies conducted in Australia and Canada, where refugees considered most likely to successfully resettle are preferentially accepted [80], reported rates of mental disorders that were lower than that of the native-born population [11, 12]. Studies conducted in the USA tended to report the highest rates of mental disorders, but also tended to report the largest variation across studies. However, the regional variations should be interpreted with caution because of the small number of studies from regions other than the USA.

Third, consistent predictors of the three mental health outcomes include greater exposure to both pre-migration traumatic experiences and post-migration stress. It has been suggested that post-migration factors might confound [81] or precede [82, 83] the impact of the prior trauma. Yet, most of the studies included in this review reported an independent effect of war trauma exposure on current mental health status even after post-migration factors were taken into account, a finding that suggests a significant and lasting impact of war experience. The impact of poor post-migration socio-economic situation, such as unemployment, financial stress, poor host language proficiency and lack of social support, was particularly evident for depression. However, considering the retrospective nature of the included studies, it cannot be decided whether a poor socio-economic status after migration is a contributing factor in the occurrence or maintenance of a mental disorder or a consequence of the pre-existing mental disorder or both. Nevertheless, findings from a longitudinal study of Southeast Asian refugees resettled in Canada indicate that persistent socio-economic hardship, particularly unemployment and poor host language proficiency, may predict depression even 10 years after resettlement [11]. Interestingly, the post-migration socio-economic situation had no impact on PTSD. Unlike depression, in which socio-economic disadvantages associated with migration may play a more significant aetiological role, aetiology of PTSD is understood as rooted in trauma exposure, possibly explaining the differences in the relative importance of socio-economic factors in the two disorders. There was a tendency towards a reduction of prevalence rates of depression with increasing length of time since displacement. Two longitudinal studies undertaken amongst Southeast Asian refugees during their 10-year resettlement period in North America suggest that depression levels decreased substantially during this period [11, 28]. Refugee demographic characteristics were less consistently associated with mental disorders. When gender was investigated as a moderator variable, it was found that prevalence rates of depression and unspecified anxiety in women tended to be higher than those in men. Such gender differences are observable in studies across the world [74, 84, 85]. In the case of PTSD both refugee males and females were found to be at increased risk which runs counter to the previous findings among the general population that suggest females are more likely to develop PTSD [86, 87]. Nonetheless, the finding concurs with that reported in the previous meta-analysis on war-affected populations [8]. Whilst in the general population men and women typically differ in types of traumatic experiences [87, 88], both civilian men and women may be exposed to similar traumatic events during war [30].

### Limitations

There are several important limitations to consider when interpreting the findings of the current review. Although a meta-analysis and data pooling was considered, it was decided that this step was inappropriate due to significant heterogeneity across studies. Instead, the analysis focused on investigating the reasons behind the between-study heterogeneity both through subgroup analysis and a qualitative assessment of studies. Whilst some trends were identified through the subgroup analysis, it is important to note that the residual heterogeneity within groups remained considerable. Therefore, clinical and methodological differences as well as a high level of statistical heterogeneity between studies (in part explained by clinical and methodological differences) may have compromised the validity of the result. It is misleading, for example, to consider that refugees in the USA are uniformly more likely to have depression; the likelihood is also dependant on other variables, such as country of origin, severity of pre-migration trauma, and also specific post-migration factors a refugee encounters. Better understanding of unique impact of these factors could be reached by applying a meta-regression; however, due to insufficient number of identified studies a meta-regression was not deemed feasible.

The present review focused on studies assessing mental disorders in long-settled war refugees. As such the search terms included the concept term “long-term”. Whilst this may have improved the specificity of the search, it may have also resulted in decreased sensitivity so that studies that did not report on the duration of displacement, or at least not in terms specified by the review, may have been missed. This may particularly be the case for studies focusing on risk factors rather than on prevalence rates. An extensive systematic review on depression and PTSD in refugees conducted by Steel et al. [8] did not identify any other relevant studies than already identified in the present study, suggesting that the current review identified all relevant literature.

The current review strategy may have further limited the generalizability of the findings on risk factors for mental disorders in long-settled refugees since the search terms do not include term the “risk factor” or related keywords. The inclusion of the term “long-term” may have limited results only to the studies that focus on impact of time as a risk factor for mental disorders. Furthermore, relying on mostly cross-sectional prevalence studies to identify risk factors for mental disorders introduces the potential for prevalence-incidence (Neyman) bias [89]. Cross-sectional studies identify prevalent rather than incident cases and the data will, therefore, tend to reflect risk factors of survival/chronicity of disorder as well as aetiology. Therefore, risk factors identified in the present review are likely to be the risk factors for maintenance of mental disorders, rather than their development.

As with any systematic review, publication bias is a potential source of error. An attempt was made to reduce the possibility of such bias by performing a thorough search strategy and by reviewing studies published in languages other than English as well as unpublished studies; however, it is still possible that some studies were not identified. There was no indication of publication bias when tested statistically, although the evidence for unspecified anxiety disorder was inconclusive. Given the large degree of heterogeneity across studies and the relatively small number of studies, non-significant results may be due to low statistical power and cannot be taken as evidence that publication bias is absent [90–93]. Nevertheless, publication bias may be less prevalent for meta-analytic reviews focusing on observational studies (as was the case in this review) than it is for randomized controlled trials [94]. Additionally, multiple tests were performed within the subgroup analyses inflating Type I error.

As already stated, at present there is no consensus on how to assess either the quality of observational studies or the impact of the study quality on the meta-analysis results [24, 25]. Similar to a previous review of mental health of refugees [9], it was decided to use a cumulative quality score incorporating methodological components common to observational studies in culturally diverse populations, with all components being given equal weight. This has the disadvantage that studies with very different level of strengths or limitations may receive similar quality scores, i.e. studies with only few, but very important limitations, may still be ranked among the higher quality studies. Nevertheless, the finding that the study quality score was associated with outcomes in the sub-group analysis does indicate that one or more components in the study quality score are associated with the outcome. This was confirmed by the results of the subgroup analysis of key quality components linked to outcomes in this and previous meta-analytic reviews of refugee mental health [2, 8].

A vote-counting method was used to assess the relationship between risk factors and mental disorders. Whilst this method provides an overall summary of the direction of the effect, it does not consider the effect size and the precision of estimated effects.

The diagnostic criteria were not consistent across studies as both structured diagnostic interviews and self-report questionnaires were used. Although the self-report questionnaires are based on and generally validated against DSM-IV diagnostic criteria [95], one has to be cautious when comparing the results. It has been argued that self-report questionnaires tend to err on the side of high sensitivity rather than high specificity, and tend to over-estimate the prevalence of psychiatric disorders [96, 97]. The most recent meta-analysis of the studies assessing prevalence of depression and PTSD in war-affected populations (including refugees) showed a pattern in which self-report questionnaires on average returned a 10–13 % higher prevalence than structured diagnostic interviews when other methodological factors were taken into account [8]. On the other hand, diagnostic instruments are based on Western concepts of illness and may be less sensitive to constellations of symptoms experienced by refugees mainly originating from non-Western cultures [98, 99]. It may be just as important not to overestimate the prevalence of mental disorders of refugee populations as it is to avoid their underestimation and subsequent underestimation of service needs.

The lack of reliable and valid instruments for use in refugee populations has often been noted. In their review of instruments used to assess mental health of refugees, Hollifield et al. [96] concluded that the majority of studies have used instruments that have limited or untested reliability and validity in refugee populations being studied. In the current review, less than 20 % of the assessment instruments identified were developed specifically in refugee research and the remainder were developed and validated in non-refugee research and, later, either adapted for and tested in refugee research or simply translated. Almost 40 % of the studies had no reliability or validity data reported at all, whilst just over a half tested instruments’ reliability and/or validity in the sample being observed. However, even if established as reliable and valid, “non-refugee” instruments are limited to Western conceptualization of mental disorders and may still not accurately reflect full range of symptoms, their meaning and perceived distress in “non-Western” cultures [96]. Coyne and Kagee [100], warn that the use of instruments with unknown validity in post-conflict populations might lead to inaccurate conclusions about the mental health burden in these populations and might even lead to erroneous decisions concerning the distribution of scarce resources. This review highlights the need for improvement of development, use, and reporting of reliable and valid instruments used to assess refugee mental health.

This review may also have lacked specificity regarding anxiety disorders, since it was decided to include studies reporting on unspecified anxiety. As already described, this outcome is one of the most frequently reported outcomes in refugees. Numerous studies have indicated that it is a valid method of identifying anxiety caseness, most strongly corresponding to the DSM-IV caseness of GAD, and to somewhat lesser extent of other anxiety disorders. Therefore, it is important to note that there is a large overlap between the findings for this outcome and PTSD, as well as other anxiety disorders.

The review included only quantitative studies. Whereas such an approach has provided an understanding of the extent of mental disorders and associated risk factors in refugees, the findings are largely based on the Western notion of mental disorders. Considering the above-noted challenges with cross-cultural understanding, interpretation, and translation in assessing refugee mental health, inclusion of qualitative studies may have helped to identify and better understand culture-specific idioms of distress and conceptions of mental health, which were not included in the existing assessment instruments. Inclusion of these studies may have also provided important insights into the trajectory of long-term mental health and the roles of individual, family and community contextual risk and protective factors in influencing the course of mental health in refugees. Such an approach may have also identified other risk and protective factors deemed important to observed refugee populations but not necessarily identified in quantitative analyses. This information is important, whether the goal is to assess trauma-related disorders in a certain cultural context or to provide culturally sensitive care [101]. However, inclusion of qualitative studies was deemed beyond the scope of this review, and this is an area in which future investigation is warranted.

The extrapolation of the findings is further precluded by several refugee selection factors. Although most studies used probability sampling, the sampling frame was necessarily opportunistic (e.g. health screening records), precluding the generalisability of findings to other refugees. The majority (86 %) of the world’s refugees currently reside in developing countries (mostly Asia and Africa); yet, most (86 %) of the studies identified by this review were conducted in Western developed countries, particularly the USA which hosts less than 2 % of the world’s refugees [1]. It is, therefore, the countries with the least resources and the most vulnerability to their security and capacity, which carry the greatest responsibility for refugee protection [102]. This presents a significant number of problems as developing countries are struggling with low resources and incoming refugees are perceived as a burden on their already weak economies. Typically, refugees in developing countries remain living in mass camp settings for decades with no prospects for durable solutions, such as repatriation or integration into local society [1]. Whilst the research on refugees in developed Western countries is valuable, applicability of its findings to the majority of refugee population is limited. This is particularly evident considering the importance of post-migration socio-economic factors, such as integration and employment, in the mental health of refugees. For refugees, who are already depleted of socio-economic resources and living in camps that are often rife with poverty, violence, and crime, the refugee experience may be very different to that of a refugee living in a developed country. Since the majority of refugees are living in poorly resourced countries, developed Western countries need to take responsibility and contribute towards burden-sharing and durable solutions for refugees. Furthermore, if effective mental health intervention strategies are to be developed, they need to take into considerations a local context of the majority of refugees, which often involves scarce resources for health and social care. Mental health interventions that truly help refugee communities in developing countries cannot succeed if reliable data on mental health needs and effective intervention strategies are not available. The lack of research in these countries indicates numerous methodological, practical and funding challenges affecting research in developing settings. Some of the identified challenges include limited local resources and skills to conduct research and difficulties in publishing their research in indexed journals due to limited access to information and advice on research design and analyses, language barrier, material and financial limitations, and policy constraints [103]. Most of these problems can be addressed through research capacity building and training, with support from or in collaboration with various stakeholders from developed Western countries [103].

It is also unclear which subgroup of refugees actually stays in a host country for more than 5 years. It may be that those with mental disorders are less motivated or able to return to the country of origin or they may even have a better chance of being allowed to stay due to having a mental illness [104]. Conversely, those without a mental disorder may be more likely to integrate and make a life for themselves in the new country and may be, therefore, more likely to remain in the country. Thus, the differences found in prevalence rates between studies may reflect different selection processes in the host countries over many years. Furthermore, over two-thirds of participants studied were from Southeast Asian countries (Vietnam, Cambodia, and Laos). Thus, the regional variations should be interpreted with caution because of the small number of studies conducted with refugees originating from regions other than Southeast Asia and residing in regions other than the USA. Focusing on the specific refugee population in well-to-do Western countries precludes generalization of prevalence rates to the majority of the worldwide refugee population. This underscores the need for research among different world refugee populations residing in diverse social, economic and political contexts.

## Conclusion

In summary, there is an obvious need for more methodologically consistent and rigorous research on the mental health of long-settled war refugees (especially those residing in developing countries). Nevertheless, substantial evidence already exists. It suggests that mental disorders tend to be highly prevalent in war refugees many years after the war experience and resettlement. Two consistent risk factors predicting higher rates of mental disorders have emerged from the cumulative body of research: past traumatic experience and the post-migration socio-economic situation.

Whilst preventing war trauma inflicted on refugees may be beyond the control of recipient countries, they can influence the post-migration challenges faced by incoming refugees by improving resettlement policies and their effectiveness in promoting long-term mental health of refugees. In terms of clinical implications, war exposure and migration remain a risk for mental disorders for many years. There is a need for treatment services for a considerable minority of refugees even many years after the resettlement. High rates of mental disorders may warrant screening programmes in primary care to identify those in need of treatment. Interventions for at risk groups may include both existing evidence-based health care interventions and psychosocial interventions. Particularly for depression, there may have to be an emphasis on managing social factors and employment schemes.
